# An Engineered Cholesterol Oxidase Catalyses Enantioselective Oxidation of Non‐steroidal Secondary Alcohols

**DOI:** 10.1002/cbic.202200075

**Published:** 2022-02-22

**Authors:** Rachel S. Heath, Jack J. Sangster, Nicholas J. Turner

**Affiliations:** ^1^ Department of Chemistry University of Manchester Manchester Institute of Biotechnology 131 Princess Street M1 7DN Manchester UK

**Keywords:** alcohols, biocatalysis, ketones, oxidation, selectivity

## Abstract

The enantioselective oxidation of 2° alcohols to ketones is an important reaction in synthetic chemistry, especially if it can be achieved using O_2_‐driven alcohol oxidases under mild reaction conditions. However to date, oxidation of secondary alcohols using alcohol oxidases has focused on activated benzylic or allylic substrates, with unactivated secondary alcohols showing poor activity. Here we show that cholesterol oxidase (EC 1.1.3.6) could be engineered for activity towards a range of aliphatic, cyclic, acyclic, allylic and benzylic secondary alcohols. Additionally, since the variants demonstrated high (*S*)‐selectivity, deracemisation reactions were performed in the presence of ammonia borane to obtain enantiopure (*R*)‐alcohols.

## Graphical abstract

**Libraries of cholesterol oxidase** variants were screened for activity towards secondary alcohols. The active variants oxidised a range of secondary alcohols including unactivated alcohols, and allylic and benzylic alcohols. In conjunction with a non‐selective reducing agent, they effected the deracemisation of racemic alcohols to yield (*R*)‐alcohols in high enantiomeric excess.

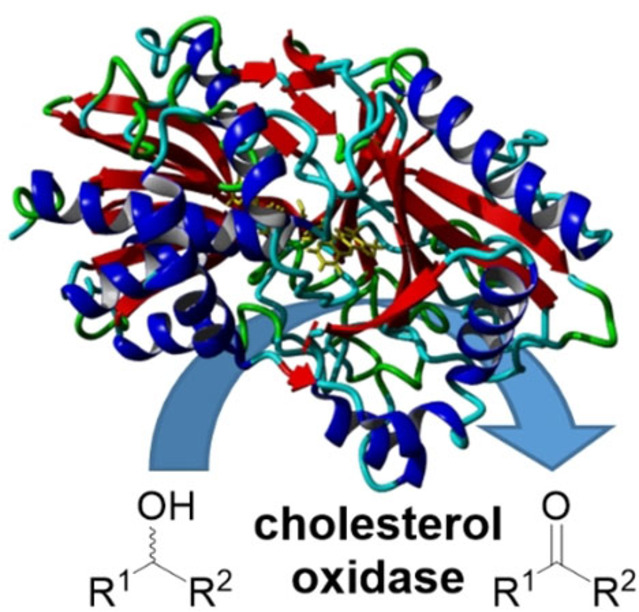

For the biocatalytic oxidation of alcohols to aldehydes and ketones, alcohol oxidases offer the following attractive properties: i) they are O_2_ driven, requiring no additional cofactor or recycling system, ii) they are amenable to protein engineering,[^1^, ^2^, ^3^, ^4^, ^5^, ^6^, ^7^, ^8^, ^9^, ^10^] iii) they can be applied in multi‐enzyme cascade reactions[^11^, ^12^, ^13^] and iv) they benefit from immobilisation.[^14^, ^15^, ^16^] All of these features were recently demonstrated in the biocatalytic synthesis of the HIV treatment drug Islatravir by the Merck group, where an evolved, immobilised, galactose oxidase variant was applied as part of a nine enzyme, three step cascade.^[11]^

The oxidation of primary alcohols to aldehydes can be achieved by a number of different alcohol oxidases and recently we engineered a primary alcohol oxidase (choline oxidase) for broadened substrate scope, increased thermostability and enhanced solvent tolerance.^[2]^ Choline oxidase is also able to oxidise diols to dialdehydes but has no activity towards secondary alcohols. Thus we were interested in exploring other alcohol oxidases for the oxidation of secondary alcohols. Previous studies of secondary alcohol oxidation by alcohol oxidases have largely focussed on activated secondary allylic or benzylic alcohols.[^1^, ^7^, ^17^, ^18^] There are some limited reports of oxidation of aliphatic secondary alcohols, in some cases alongside their enantioselectivity, although the substrate scope is narrow and it is not clear how these biocatalysts might perform more generally for synthetic applications.[^19^, ^20^, ^21^, ^22^]

We targeted cholesterol oxidase (EC 1.1.3.6) in order to develop a broad spectrum biocatalyst for the oxidation of secondary alcohols. Cholesterol oxidases have found application in the assay and detection of cholesterol in blood serum and have been used in many biosensors; in agriculture they have been used as insecticidal agents against weevil larvae, and they also play a role in the synthesis of the antifungal antibiotic pimaricin.^[23]^ As well as oxidising cholesterol (Figure 1a), cholesterol oxidase oxidises a range of steroidal substrates,^[23]^ and the cholesterol oxidase from *Rhodococcus erythropolis* was also found to oxidise the non‐steroidal substrates 2‐cyclohexen‐1‐ol **4** and 3‐methyl‐2‐cylohexen‐1‐ol **5** with (*S*)‐selectivity at the reaction centre (Figure 1b).[^24^, ^25^] Benzyl alcohol, isopropanol and cyclohexanol were reportedly substrates of cholesterol oxidase from *Streptomyces hygroscopicus* but only as indicated by the (very slow) disappearance of the oxidized flavin band in the visible spectrum.^[26]^ This led us to investigate whether we could use cholesterol oxidase for the oxidation of other secondary alcohols (Figure 1c).

**Figure 1:**
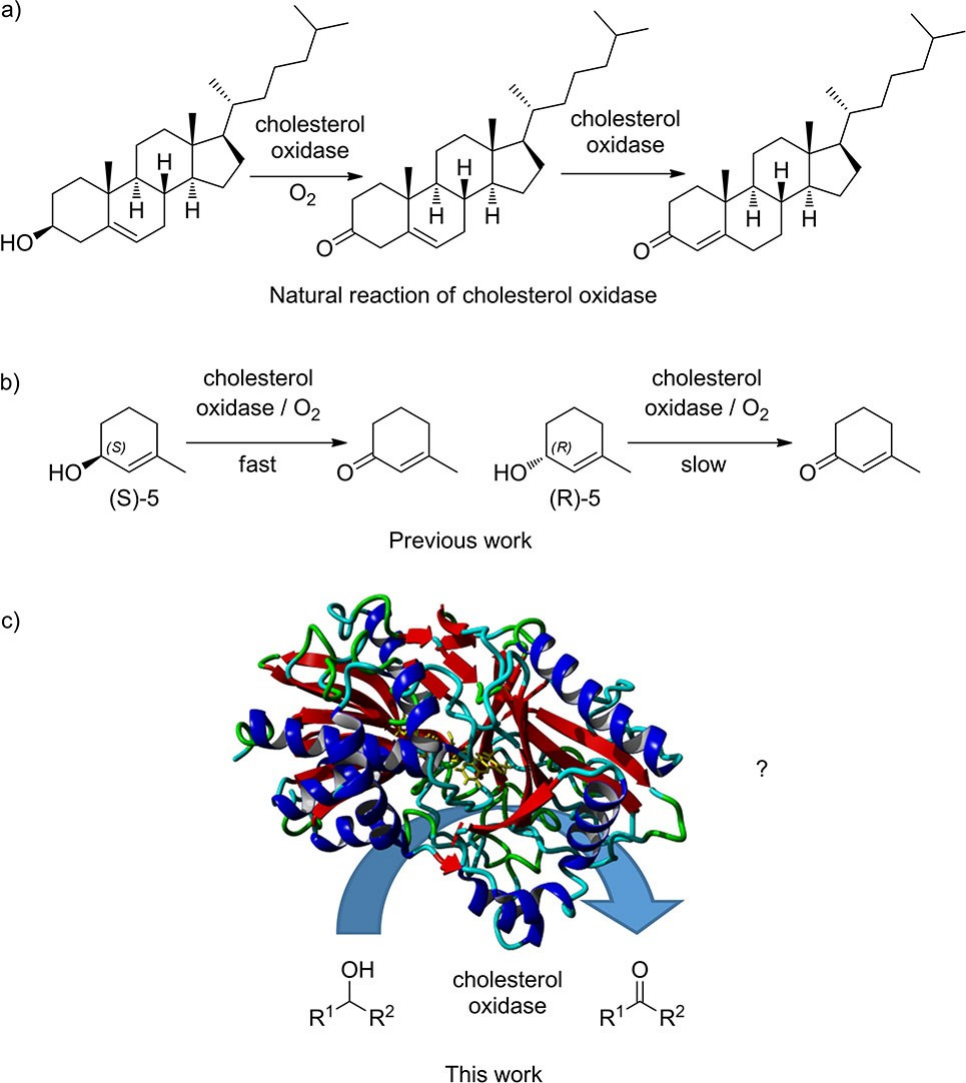
The oxidation reactions of cholesterol oxidase: a) the natural reaction of cholesterol oxidase, b) the selective oxidation of secondary alcohols by cholesterol oxidase from *Rhodococcus erythropolis*, c) the aim of this work.

Cholesterol oxidases from *Rhodococcus erythropolis* (ReCO), *Brevibacterium sterolicum* (BsCO) and *Streptomyces hygrospinosus* (ShCO) were our initial targets for investigation. Preliminary experiments revealed that ShCO could be expressed in *E. coli* and showed some activity towards 3‐methyl‐2‐cyclohexen‐1‐ol **5**. ReCO expressed poorly in *E. coli* and BsCO showed no activity towards **5** and thus we selected ShCO as our target enzyme. Initial assays with ShCO on a broader range of alcohol substrates (see Figure 3) however showed either no, or low conversion, and we thus embarked on a strategy of directed evolution to broaden the substrate scope.

Libraries were prepared encompassing the active site and entrance tunnel as shown in Figure 2a, namely F122, M165, P387, E404/P409, L418/L420, Y489/H490, F476 and N528. These site‐saturation libraries were screened using a solid‐phase assay (described in more detail in the SI), which detects the hydrogen peroxide that is produced when the enzyme oxidises the substrate. The two targets were 2‐cyclo‐hexen‐1‐ol **4**, because even though similar in structure to 3‐methyl‐2‐cyclohexen‐1‐ol **5** we detected no initial activity, and cyclohexanol **2** as this is a non‐activated cyclic alcohol for which there are few reports of good alcohol oxidase activity.

**Figure 2:**
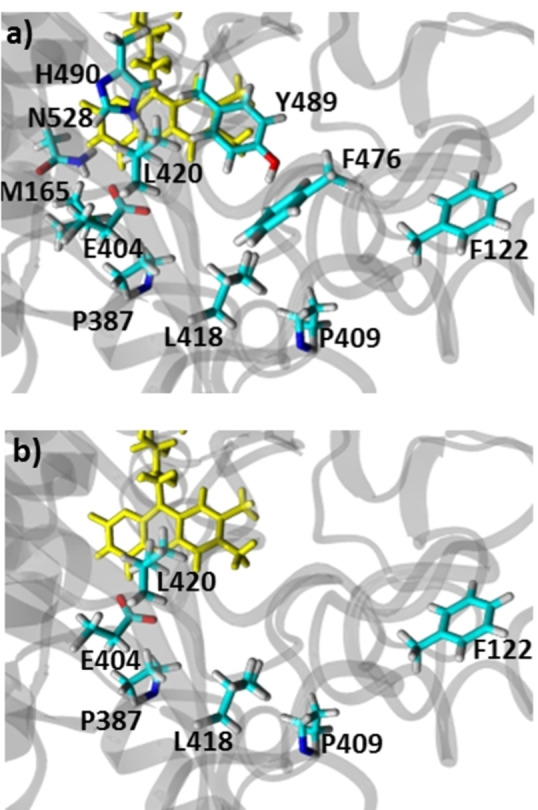
a) Sites in ShCO where libraries were prepared, b) positions where hits were found. The (non‐covalent) FAD cofactor is shown in yellow. Homology model of ShCO and the graphical figure were prepared using molecular modelling software YASARA^[27]^ (www.yasara.org) and POVRay (www.povray.org).

The sites that were screened and where hits occurred are shown in Figure 2b and described in more detail in the supporting information (Table S3). We additionally explored direct recombination of hits: For example, we combined the hit E404C/P409S with the hit P387W but this led to a decrease in conversion from 99 % to 25 % as measured by GC‐FID (data not shown). We also tried an iterative approach where we started with an active variant and used that as the background to make further libraries in. For example, we used the variant E404C/P409S and made library P387 but this gave no hits when screened with the solid‐phase screen, (Further details of the combinations tried are described in the Results and Discussion section of the SI). In the end, the most active variant for both substrates was found to be a two point variant from the E404/P409 library. For 2‐cyclohexen‐1‐ol the best variant was E404C/P409S, named ShCO_a_ from hereon in, and for cyclohexanol the best variant was E404A/P409I, now referred to as ShCO_b._

We further explored the activity of our variants with other substrates as shown in Figure 3 by performing biotransformations and measuring the conversion. The variant given is the most active for that substrate, though, for substrates **3**, **7**, **8**, **9**, **11**, and **17** both variants showed similar conversions. We initially compared conversions of wild‐type and variants at 10 mM substrate concentration (Figure 3, a) but later determined that the ShCO variants maintained good conversions at 50 mM (Figure 3, b). For cyclohexanol **2** and hexanol **17**, conversions with 100 mM substrate were obtained (86 % and 58 % respectively) showing potential for use of the enzyme at higher substrate loadings.

**Figure 3:**
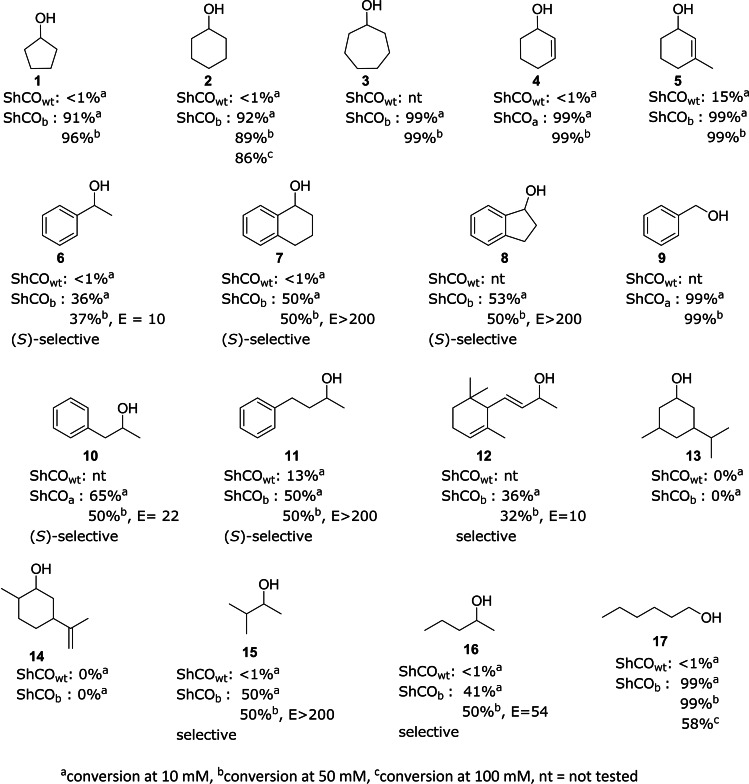
Substrate scope of cholesterol oxidase variants. Conversion and selectivity data are shown for conversion of the substrate to the respective ketone or aldehyde product at the substrate concentration defined by superscript ^a,^ ^b^ or ^c^. Conditions: 100 mM air‐saturated potassium phosphate buffer, pH 8; 17 μM purified enzyme; 30 °C; 180 rpm; 24 h. Biotransformations were extracted into tert‐butyl methyl ether and conversions and selectivity determined by GC‐FID or HPLC.

The ShCO variants were active on a range of aliphatic, cyclic, acyclic, allylic and benzylic alcohols. In most cases, either full conversion was achieved, or 50 % conversion where the enzyme was selective. Despite a similarity in structure to the other molecules, **13** and **14** were not substrates. Modelling of **13** into the active site of a homology model of ShCO_b_ suggests that there is a steric clash between the FAD and the isopropyl/isopropenyl group at the C3 position of **13/14** (Figure S19).

We could show that the primary alcohols, 1‐hexanol and benzyl alcohol, were also excellent substrates for the variants, and thus the enzyme was not restricted to secondary alcohols (Figure 3). We also examined the kinetics of hexanol **17** and cyclohexanol **2** (Table S4, Figure S17). Although ShCO_a_ and ShCO_b_ both show good activity for 1‐hexanol, the kinetic studies demonstrate that variant ShCO_b_ shows a higher rate of oxidation of substrate **17**. The K_M_ values for wild‐type cholesterol oxidase with cholesterol as a substrate are in the 1–100 μM range.^[28]^ In comparison, the K_M_ values here were in the range of 10–100 mM, in keeping with the promiscuity of the variants and their ability to catalyse reactions with substrates in that concentration range with good conversion.

Generally, the enzyme showed excellent enantioselectivity with many E values being >200 (accurate values above 200 cannot be determined due to inaccuracies in the ee value determination). Substrates **6** and **12** were mediocre substrates for the enzyme with lower conversions and E values of 10. Although substrates **4** and **5** are chiral, both enantiomers were converted. Biotransformations at higher substrate concentrations however showed that the enzyme still displayed selectivity for one enantiomer over the other (data not shown). For all substrates where the selectivity could be determined, the enzyme was (*S*)‐selective. For those substrates where conversion was 50 % we also performed deracemisation reactions with 50 mM racemic alcohol and the ShCO variant. For these reactions a non‐selective chemical reductant (ammonia borane) was added and the results are shown in Table 1. Good to excellent *ee* values were achieved for the majority of substrates except for **16**.To assess the stability of the enzyme we looked at thermostability and solvent tolerance. We examined the T_50_ (temperature at which 50 % of the residual activity is maintained) and determined T_50_=50.9 °C (Figure S20). This parameter is an indication of kinetic stability and is often a good estimation of the T_M_ (the temperature at which half the population of enzyme molecules are unfolded). We also ran reactions with biphasic systems (50 % v/v solvent/buffer) and showed no detrimental effect of cyclohexane, ethyl acetate, or tert‐butyl methyl ether compared to buffer (Figure 4).

**Table 1:** Results of deracemisation using ShCO variants and ammonia borane.

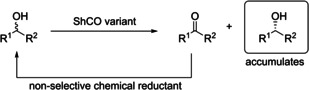				
Substrate	Enzyme	% alcohol	% ketone	% ee alcohol
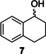	ShCO_b_	>99	0	99 (*R*)
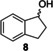	ShCO_a_	98	2	89 (*R*)
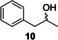	ShCO_a_	>99	0	94 (*R*)
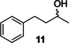	ShCO_b_	>99	0	96 (*R*)
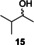	ShCO_b_	>99	0	99
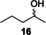	ShCO_b_	>99	0	54

Conditions: 100 mM air‐saturated potassium phosphate buffer, pH 8; 17 μM purified enzyme; 50 mM substrate; 200 mM ammonia borane; 30 °C; 180 rpm; 24 h. Conversions and selectivity determined by GC‐FID or HPLC.

**Figure 4:**
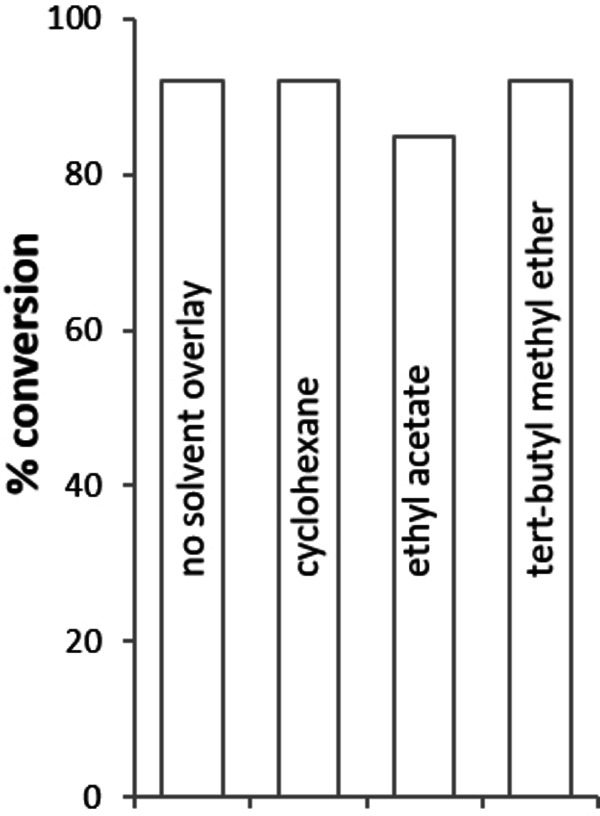
Comparison of conversion with solvent overlay compared to no solvent overlay. Conditions: 100 mM air‐saturated potassium phosphate buffer, pH 8; 17 μM purified enzyme; 90 mM 2‐cyclohexen‐1‐ol **4**; 30 °C; 180 rpm; 24 h. Conversions determined by GC‐FID.

Finally we performed preparative reactions on a 100 mg scale (Figure 5) with substrates **3**, **4** and **7** to prepare either ketones or single alcohol enantiomers (by employing the deracemisation process described above). Due to the reasonable stability of the enzymes we were able to employ a biphasic system because, in particular for substrate **7**, we were approaching the substrate solubility limit in water. We were able to demonstrate good to excellent yields for both oxidation and deracemisation reactions.

**Figure 5:**
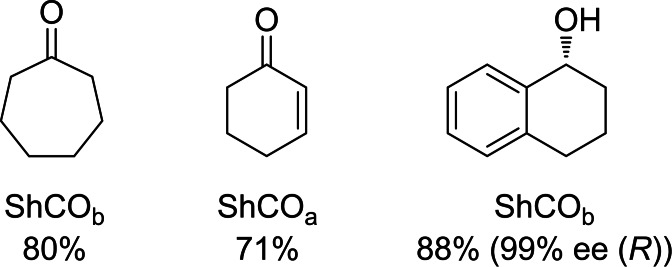
Products of oxidative and deracemisation scaled reactions are shown alongside yield and the variant used in the reaction.

In this work we have identified double mutants of cholesterol oxidase that are active on a range of non‐steroidal alcohols, including cyclohexanol and 2‐cyclohexen‐1‐ol. Although alcohol oxidase activity has previously been demonstrated for cyclohexanol, 2‐cyclohexen‐1‐ol and 1‐phenyl ethanol, conversions or selectivity were poor.[^18^, ^22^, ^29^] In contrast to our poor E value for 2‐pentanol **16**, the alcohol oxidase from *Phanerochaete chrysosporium* gives E>200.^[22]^ Our reactions all proceeded with (*S*)‐selectivity suggesting this might be an inherent property of cholesterol oxidases,^[24]^ and indeed, many other alcohol oxidases also display (*S*)‐selectivity.[^4^, ^7^, ^17^, ^18^, ^22^, ^30^] Conversely, an engineered galactose oxidase variant displayed (*R*)‐selectivity^[1]^ and has complementary selectivity for substrates **6**, **7** and **8**.

Mutation of E404 (E361) to glutamine in a homologous *Streptomyces* cholesterol oxidase showed that it was essential for the isomerisation reaction but not the oxidation reaction of cholesterol.^[28]^ Prolines introduce rigidity in a structure and mutation is often considered not advisable because of the effect it can have on the structure, either by stabilisation or destabilisation.^[31]^ Mutation of P387 also resulted in hits (see SI) but when combined with the double E404/P409 variant, the effect was detrimental. In this case then, mutation of one proline may introduce enough flexibility to allow access of alternative substrates to the active site but when two are mutated perhaps too much rigidity is lost and the active site/entrance doesn't maintain enough specificity for catalysis.

The stability of biocatalysts, including thermostability and solvent tolerance are important properties of enzymes for use in industry.^[32]^ The variants reported here showed solvent tolerance and could be used in biphasic systems for the scale‐up of reactions which suffer from poor aqueous solubility of the substrate. However, further engineering could be employed to improve the thermostability if necessary.^[33]^

In summary, we have evolved cholesterol oxidase towards acceptance of a range of secondary alcohols and demonstrated its use as a biocatalyst for oxidation and deracemisation of racemic alcohols, thus adding to the ever‐growing toolbox of alcohol oxidases for biocatalytic oxidation reactions.

## Conflict of interest

The authors declare no conflict of interest.

## Supporting information

As a service to our authors and readers, this journal provides supporting information supplied by the authors. Such materials are peer reviewed and may be re‐organized for online delivery, but are not copy‐edited or typeset. Technical support issues arising from supporting information (other than missing files) should be addressed to the authors.

Supporting Information

## Data Availability

The data that support the findings of this study are available in the supplementary material of this article.
