# Supplementary material for: An Engineered Cholesterol Oxidase Catalyses Enantioselective Oxidation of Non‐steroidal Secondary Alcohols
Source: Chembiochem. 2022 Feb 22;23(7):e202200075. doi: 10.1002/cbic.202200075 (PMC9303356; doi:10.1002/cbic.202200075)
Supplement: Supplementary file 1 — Supporting Information [file CBIC-23-0-s001.pdf]

ChemBioChem

Supporting Information

An Engineered Cholesterol Oxidase Catalyses Enantioselective Oxidation of Non-steroidal Secondary Alcohols

Rachel S. Heath, Jack J. Sangster, and Nicholas J. Turner*

Supporting Information

Table of Contents

Experimental procedures 3
1. General Chemicals 3
2. Biotransformations and analysis 3
2.1 Biotransformations 3
2.2 Analysis 3
2.2.1 GC-FID methods 3
2.2.2 HPLC method 4
2.2.3 GC-FID traces by substrate, alcohol to carbonyl conversions 4
2.2.4 Scaled reactions 19
2.2.5 NMR and optical rotation 19
3. DNA and protein methods 20
3.1 Protein sequences 20
3.2 Cloning 20
3.3 Expression and purifications 20
3.4 Libraries 20
3.4.1 Synthesis of libraries 20
3.4.2 Solid phase screen 21
3.5 Kinetics 21
3.6 Temperature and solvent stability 22
3.6.1 Biotransformations using solvents overlays 22
3.6.2 T_{50} calculations 22
3.7 Modelling 22
Results and Discussion 23
Hits from screening libraries 23
Kinetics of variants with hexanol and cyclohexanol 23
Modelling of inactive substrates 24
Temperature stability 24
References 24
Author contributions 24

Experimental Procedures

1. General chemicals

Substrates and product standards were purchased from Sigma Aldrich in the highest grade possible unless otherwise stated. (*R*)-phenyl-2-propanol was from Fluorochem, and 4-phenyl-2-butanol, 3-methyl-2-butanol, and 2-pentanone were purchased from Alfa Aesar. The source of any other reagents that were used are referred to in the relevant section below.
Solvents were either analytical or HPLC grade - tert-butyl methyl ether, ethyl acetate, and cyclohexane were purchased from Fisher Scientific.

2. Biotransformations and analysis

2.1 Biotransformations

500 µl biotransformation reactions were set up (final concentration enzyme: 17µM, final concentration substrate: 10 mM or 50 mM or 100 mM). The biotransformations were made up in air-saturated 100 mM potassium phosphate buffer, pH 8.0. Biotransformations were typically carried out in a 2 mL eppendorf tube. Substrates were added either neat or from a 1 M solution made up in DMSO (Sigma). Reactions were incubated at 30 °C, 200 rpm for 24 hrs. Deracemisations additionally contained ammonia borane (Sigma) in a four-fold excess over substrate. Biotransformations were extracted by addition of 1 mL *tert*-butyl methyl ether (TBME), vortexing for 1 min followed by centrifugation at 13000 rpm for 2 min to separate the phases. The organic phase was analysed by GC-FID.

2.2 Analysis

2.2.1 GC-FID methods

GC-FID analysis was performed on an Agilent 6850 GC with a Gerstel Multipurposesampler MPS2L. The methods used are given in Table SI2. Two columns were used for analysis: β-dex 325 (Supelco) with dimnsions 30 m x 0.25 mm x 0.25µm, and Chirasil β-dex CB (Agilent) with dimensions 25 m x 0.25 mm x 0.25µm with a detector temperature 250°C and injector temperature 250 °C. The methods used are described in Table S1 with Figure S1 showing which method was used for which substrate.

2.2.2 HPLC analysis method

HPLC analysis was performed on an Agilent system equipped with a G1379A degasser, G1312A binary pump, a G1329 autosampler unit, a G1315B diode array detector and a G1316A temperature controlled column compartment. The column used a CHIRALCEL® DAICEL OD-H (5 µm particle size, 4.6 mm diameter x 250 mm). HPLC analysis was used for compound **8** (Figure S1). The method used was: flowrate 0.8 mL / min; eluent = hexane/iPrOH 95:5; UV detection at 265 nm.

Table S1: Temperature gradients for GC-FID analyses

Method name	Column	Oven		
		ramp	*temp*	*hold*
A	Supelco beta-dex 325		110 °C	20 min
		10 °C / min	200 °C	-
B	Chirasil β-dex CB		80 °C	5 min
		5 °C / min	120 °C	-
		10 °C / min	200 °C	5 mins
C	Chirasil β-dex CB		50 °C	-
		5 °C / min	200 °C	5 min
D	Chirasil β-dex CB		40 °C	5 min
		5 °C / min	120 °C	0 min
		10 °C / min	200 °C	0 min

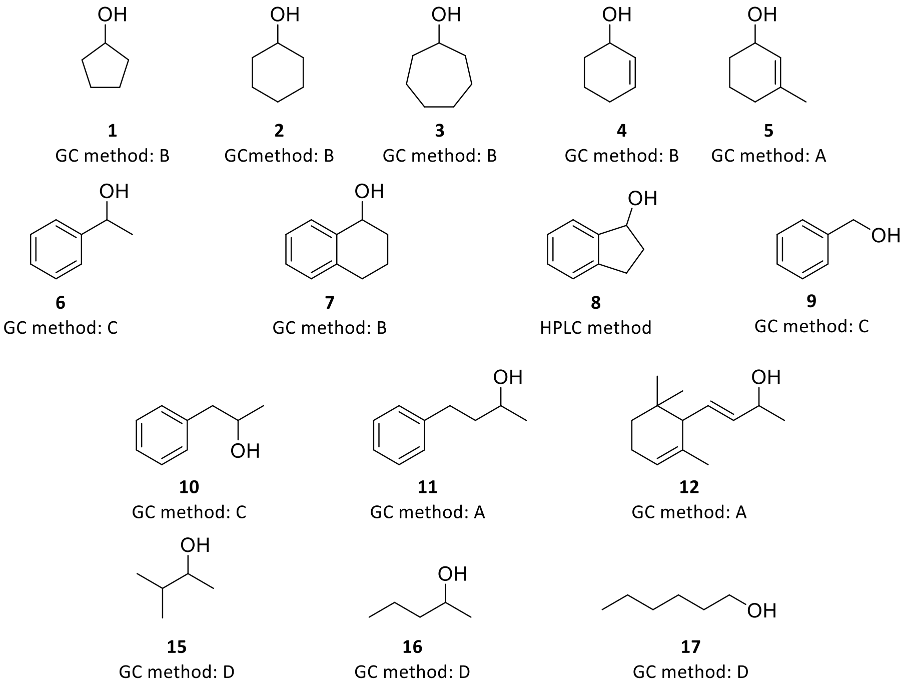

Figure S1: Substrate and method number for GC and HPLC analysis

2.2.3 GC-FID and HPLC traces by substrate, alcohol to carbonyl conversions

Note: some biotransformations with WT enzyme were recorded on an older (shortened) GC column and thus have different retention times. They are thus shown as separate spectra with substrate and product standards. LHS denotes ‘left-hand side’, RHS denotes ‘right-hand side’ in the figure labelling.

Traces for substrates 13 and 14 are not shown because there was no conversion.

*2.2.3.1 Cyclopentanol (**1**)*

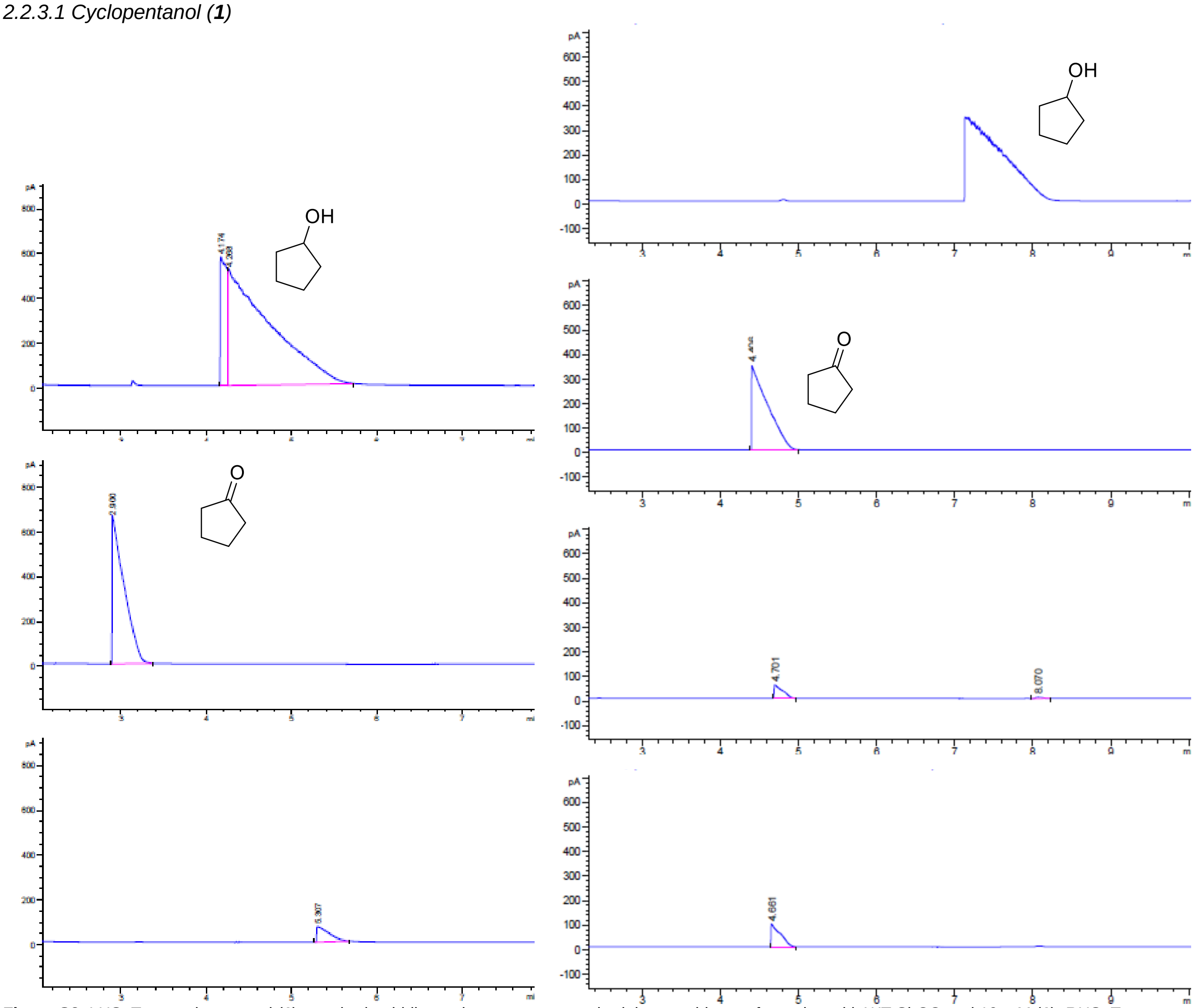

Figure S2: LHS: Top: cyclopentanol (**1**) standard; middle: cyclopentanone standard; bottom: biotransformation with WT ShCO and 10 mM (**1**). RHS: Top: cyclopentanol (**1**) standard; 2nd: cyclopentanone standard; 3rd: biotransformation with ShCO$_b$ and 10 mM (**1**); bottom: biotransformation with ShCO$_b$ and 50 mM (**1**).

*2.2.3.2 Cyclohexanol (**2**)*

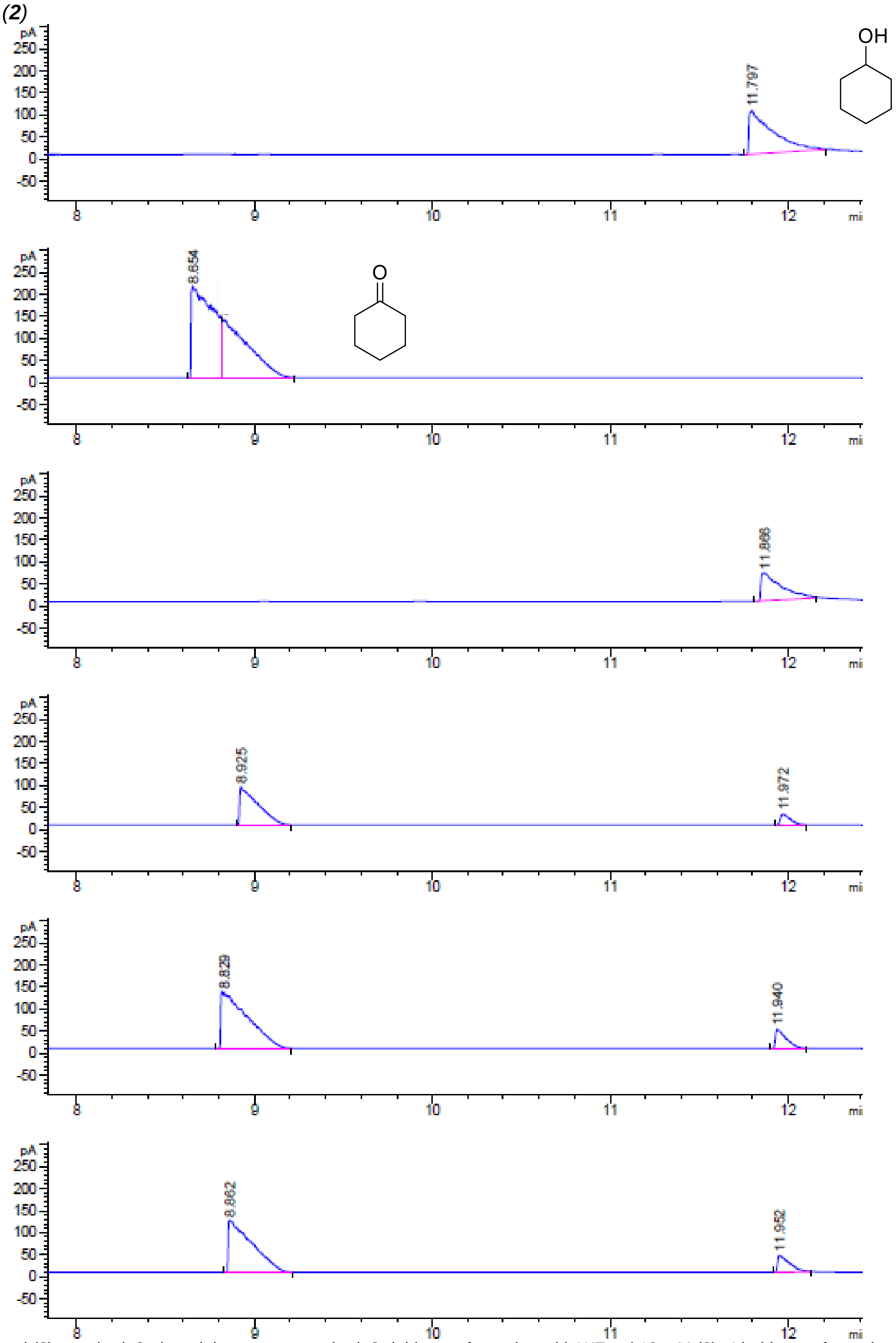

Figure S3: Top: cyclohexanol (**2**) standard; 2nd: cyclohexanone standard; 3rd: biotransformation with WTand 10 mM (**2**); 4th: biotransformation with ShCO$_b$ and 10 mM (**2**); 5th: biotransformation with ShCO$_b$ and 50 mM (**2**); bottom: biotransformation with ShCO$_b$ and 100 mM (**2**).

*2.2.3.3 Cycloheptanol (**3**)*

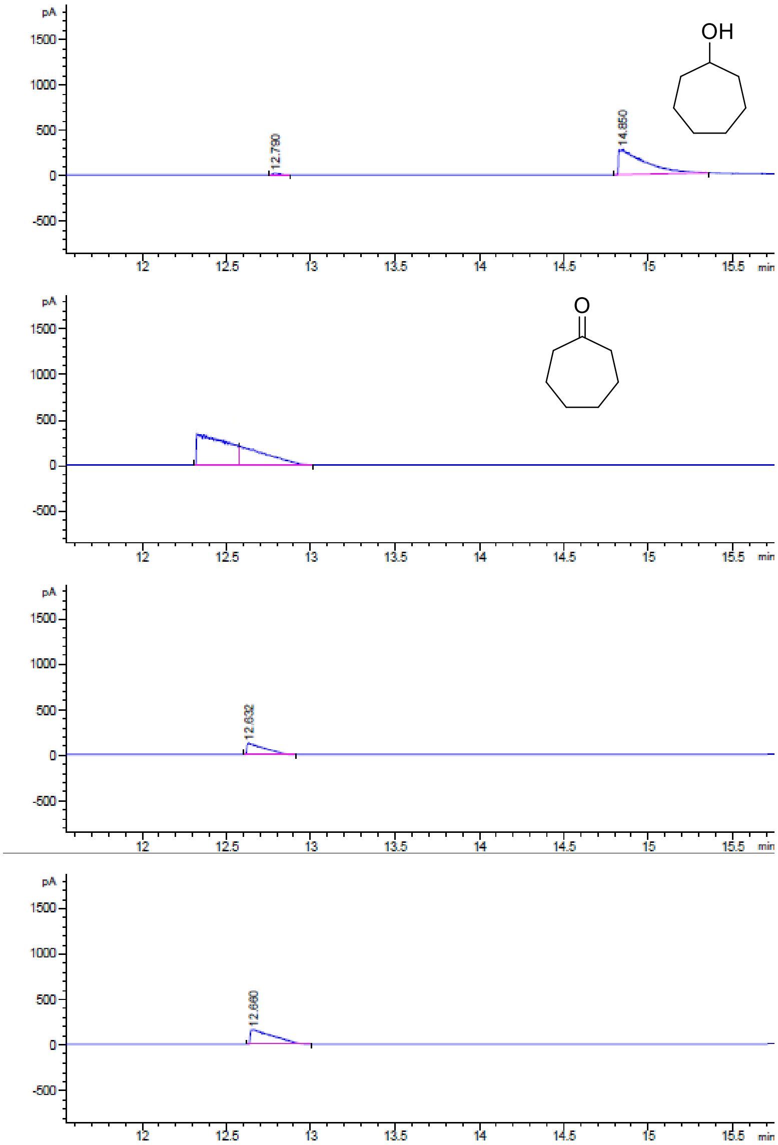

Figure S4: Top: cycloheptanol (**3**) standard; 2nd: cycloheptanone standard; 3rd: biotransformation with $ShCO_b$ and 10 mM (**5**); bottom: biotransformation with $ShCO_b$ and 50 mM (**3**).

*2.2.3.4 2-cyclohexen-1-ol (**4**)*

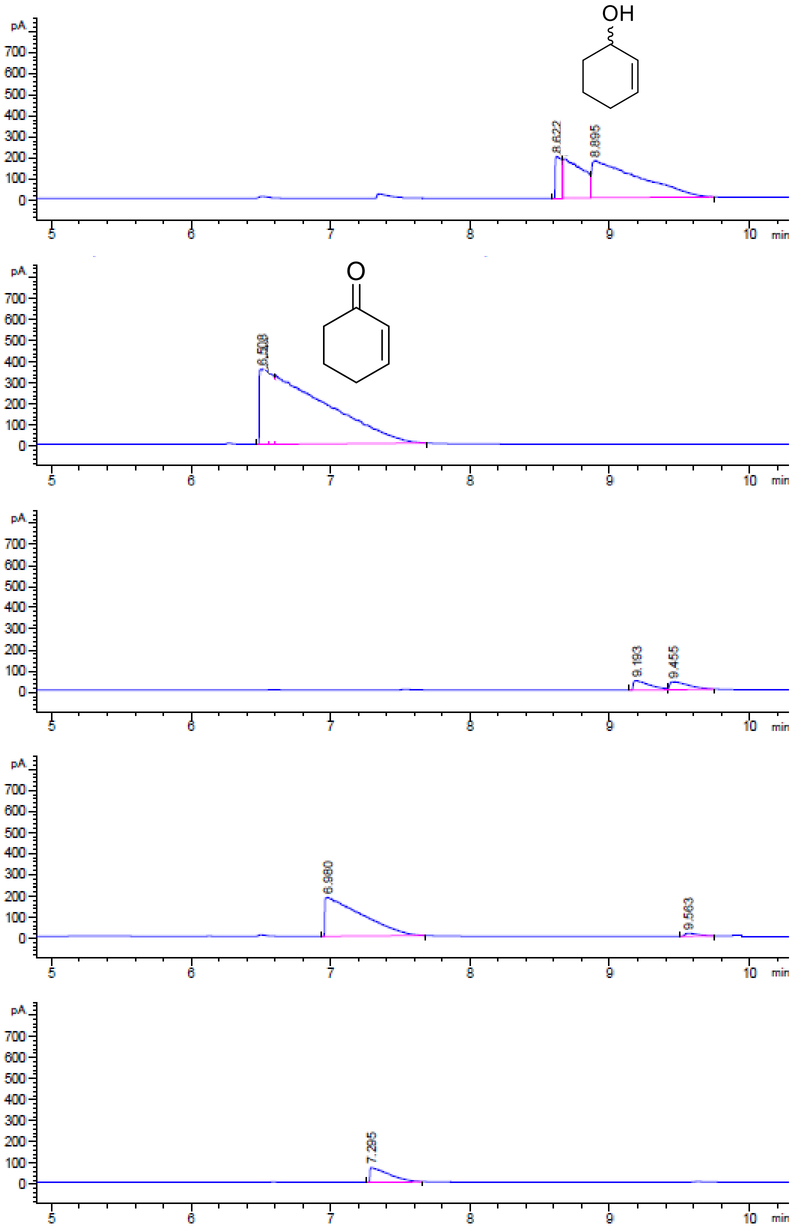

Figure S5: Top: 2-cyclohex-2-en-1-ol (**4**) standard; 2nd: 2-cyclohex-2-en-1-one standard; 3rd: biotransformation with WTand 10 mM (**4**); 4th: biotransformation with $ShCO_a$ and 10 mM (**4**); bottom: biotransformation with $ShCO_a$ and 50 mM (**4**).

*2.2.3.5 3-methylcyclohex-2-en-1-ol (**5**)*

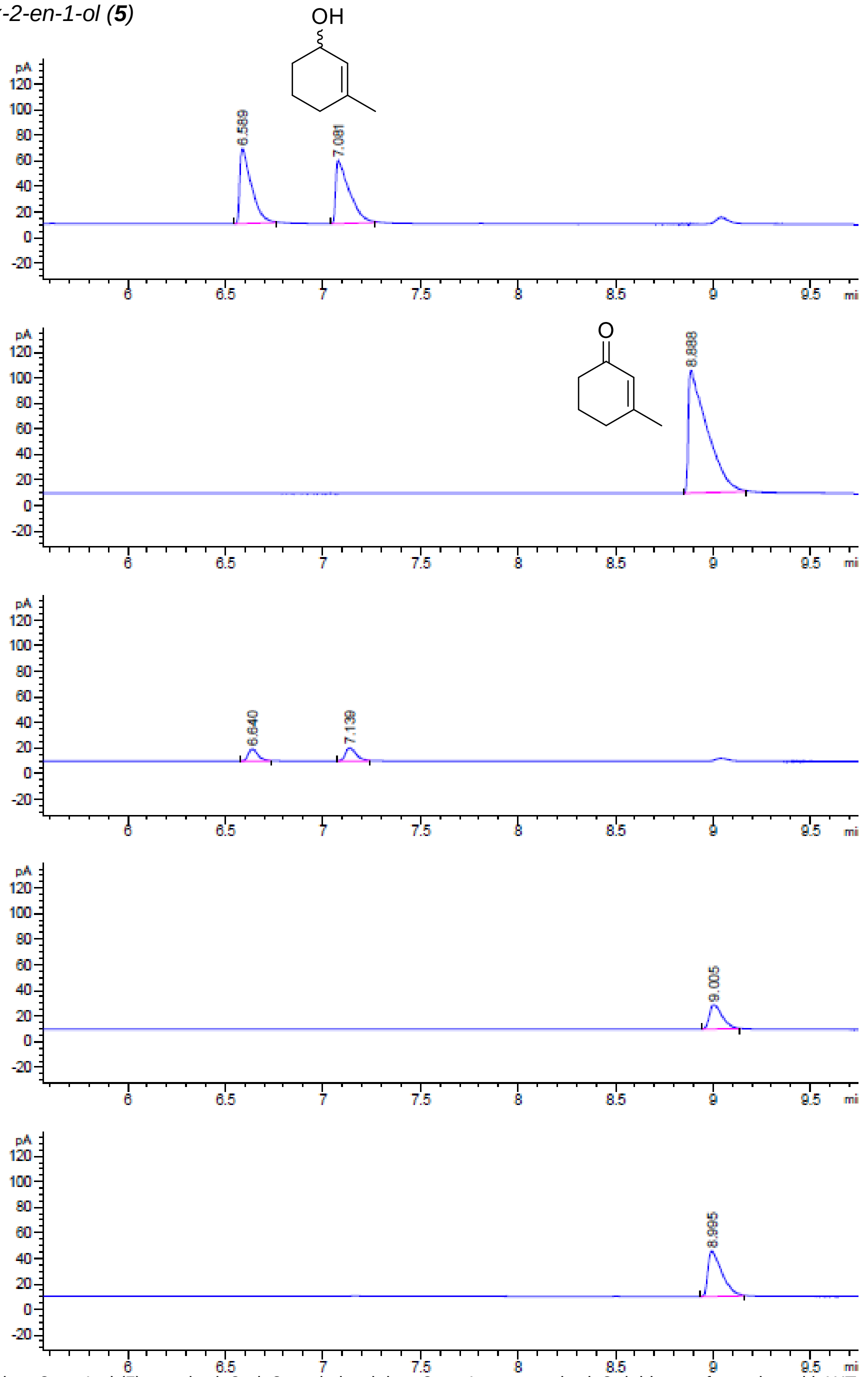

Figure S6: Top: 3-methylcyclohex-2-en-1-ol (**5**) standard; 2nd: 3-methylcyclohex-2-en-1-one standard; 3rd: biotransformation with WTand 10 mM (**5**); 4th: biotransformation with $ShCO_b$ and 10 mM (**5**); bottom: biotransformation with $ShCO_b$ and 50 mM (**5**).

*2.2.3.6 1-phenylethan-1-ol (**6**)*

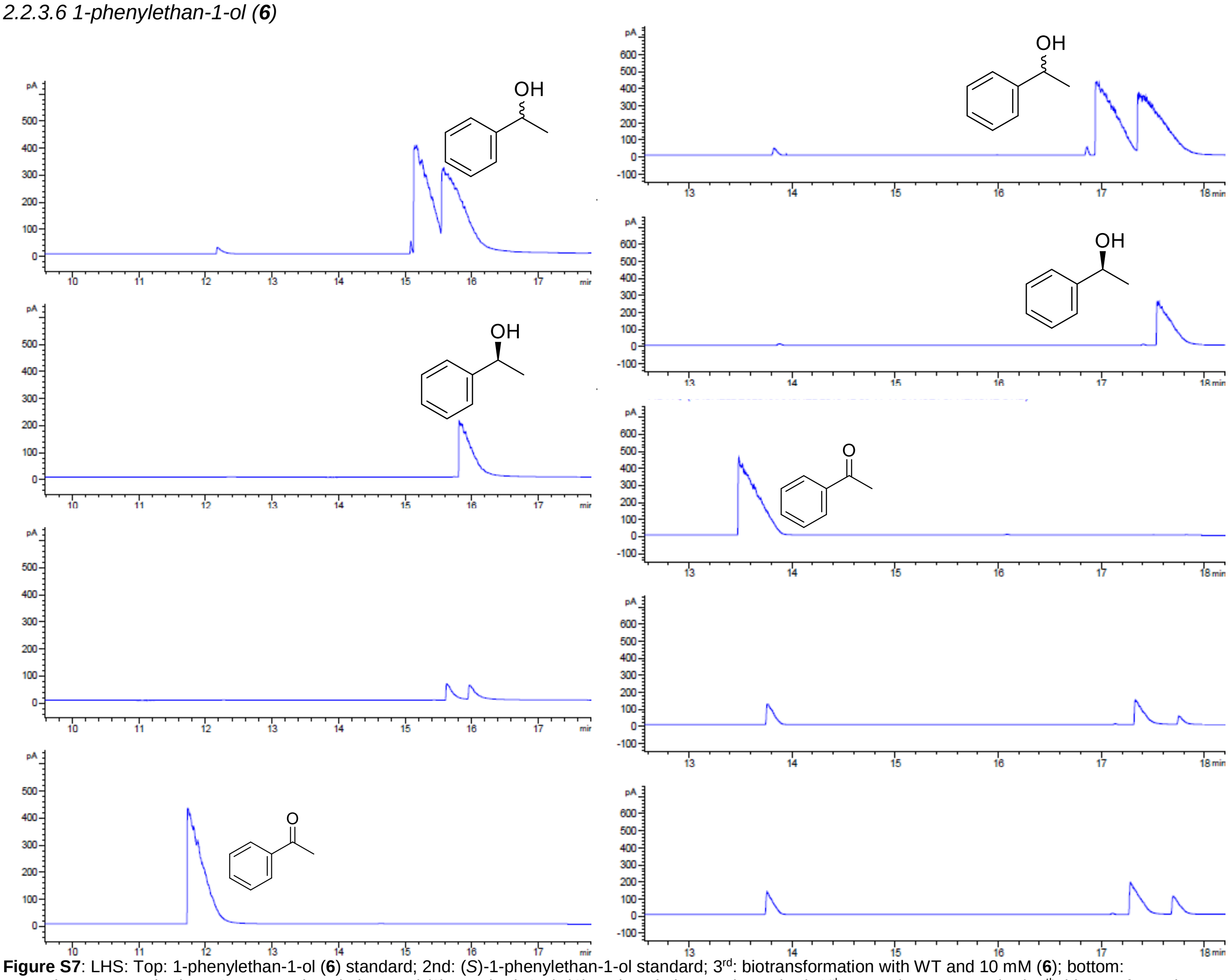

Figure S7: LHS: Top: 1-phenylethan-1-ol (**6**) standard; 2nd: (*S*)-1-phenylethan-1-ol standard; 3rd: biotransformation with WT and 10 mM (**6**); bottom: acetophenone standard. RHS: Top: 1-phenylethan-1-ol (**6**) standard; 2nd: (*S*)-1-phenylethan-1-ol standard; 3rd: acetophenone standard; 4th: biotransformation with $ShCO_b$ and 10 mM (**6**); bottom: biotransformation with $ShCO_b$ and 50 mM (**6**).

*2.2.3.7 1,2,3,4-tetrahydronaphthalen-1-ol (**7**)*

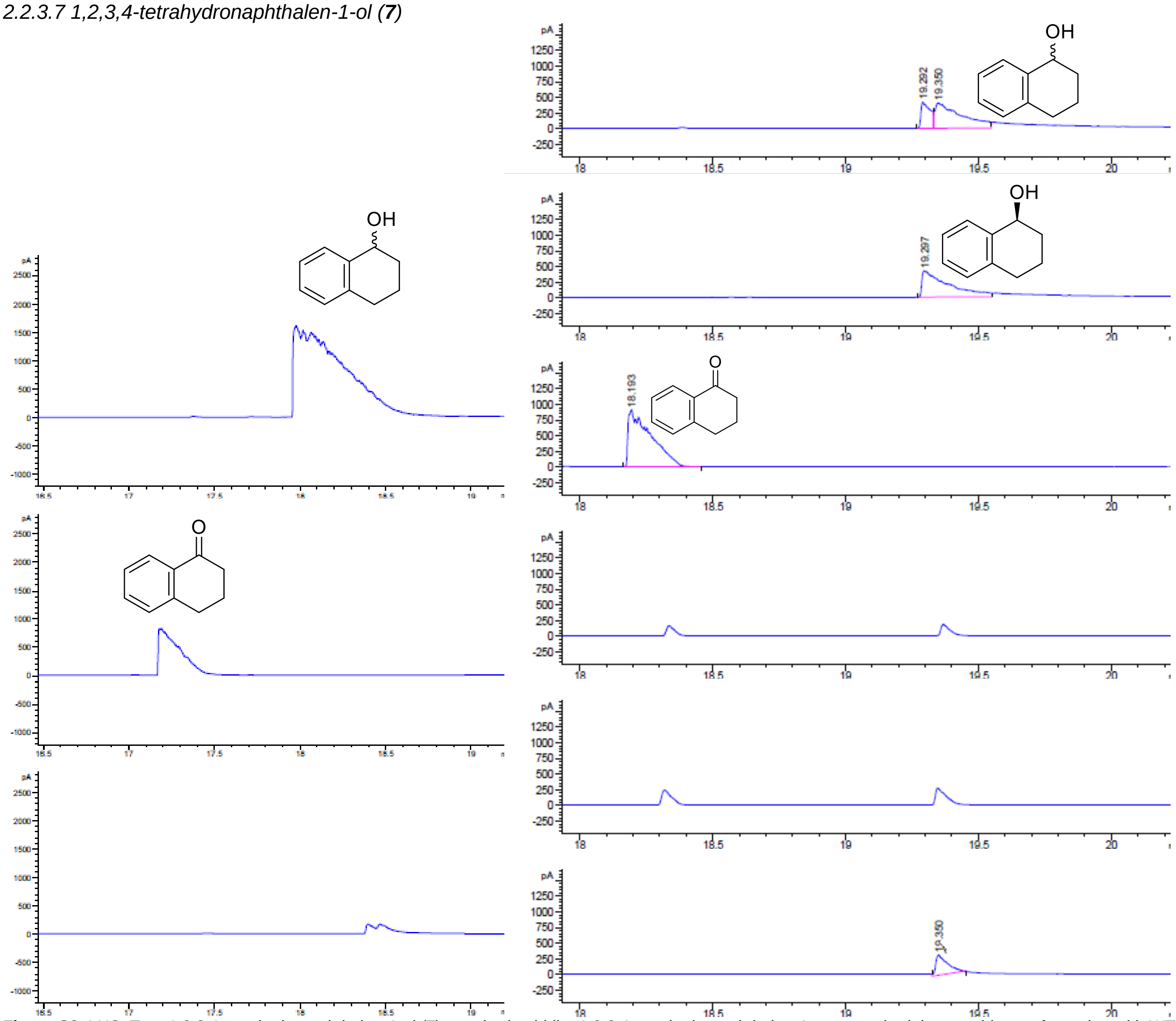

Figure S8: LHS: Top: 1,2,3,4-tetrahydronaphthalen-1-ol (**7**) standard; middle: 1,2,3,4-tetrahydronaphthalen-1-one standard; bottom: biotransformation with WT ShCO and 10 mM (**7**). RHS: Top: 1,2,3,4-tetrahydronaphthalen-1-ol (**7**) standard; 2nd: (*S*)-1,2,3,4-tetrahydronaphthalen-1-ol standard; 3rd: 1,2,3,4-tetrahydronaphthalen-1-one standard; 4th: biotransformation with ShCO$_b$ and 10 mM (**7**); 5th: biotransformation with ShCO$_b$ and 50 mM (**7**); bottom: deracemisation with ShCO$_b$ and 50 mM (**7**) and ammonia borane. Rt enantiomers = 19.29 min, 19.35 min. Rt (*S*)-enantiomer = 19.29 min, Rt deracemisation product = 19.35 min = (*R*) enantiomer.

*2.2.3.8 1-indanol (**8**)*

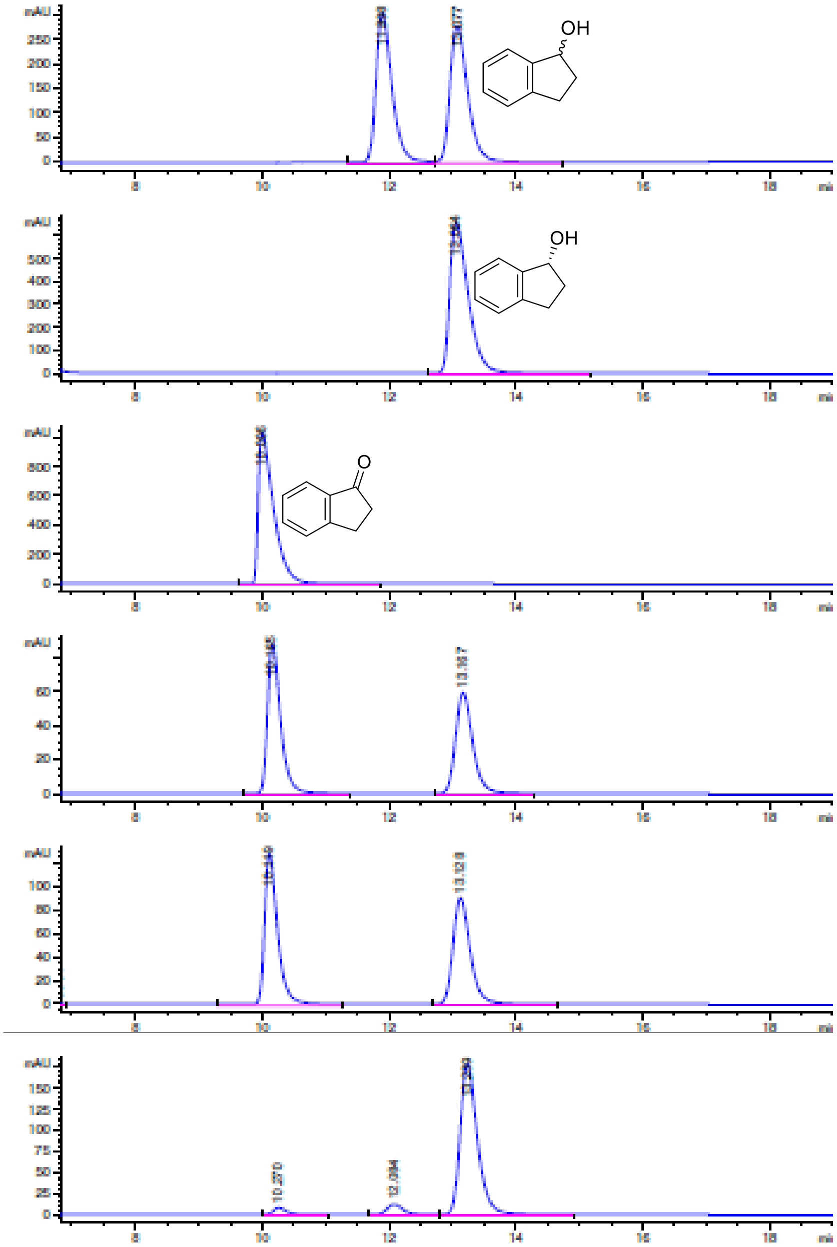

Figure S9: Top: 1-indanol (**8**) standard; 2nd: (*R*)-indanol standard; 3rd: indanone standard; 4th: biotransformation with $ShCO_b$ and 10 mM (**8**); 5th: biotransformation with $ShCO_b$ and 50 mM (**8**); bottom: deracemisation with $ShCO_b$ and 50 mM (**8**) and ammonia borane. Rt enantiomers = 11.9 min (*S*), 13.1 min (*R*), Rt deracemisation product = 13.1 min = (*R*) enantiomer

.

*2.2.3.9 Benzyl alcohol (**9**)*

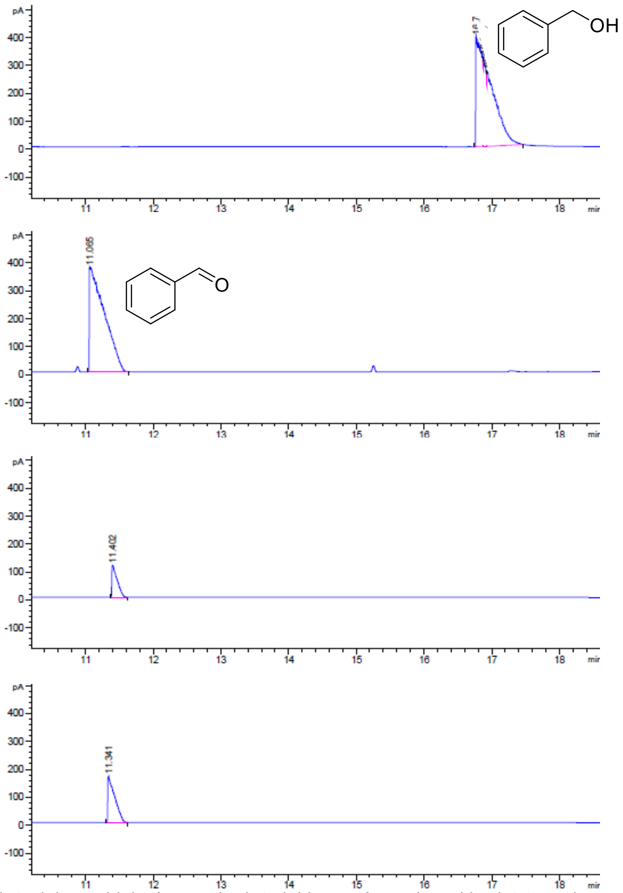

Figure S10: Top: benzyyl alcohol (**9**) standard; 2nd: benzaldehyde standard; 3rd: biotransformation with $ShCO_a$ and 10 mM (**9**); bottom: biotransformation with $ShCO_a$ and 50 mM (**9**).

*2.3.3.10 1-phenylpropan-2-ol (**10**)*

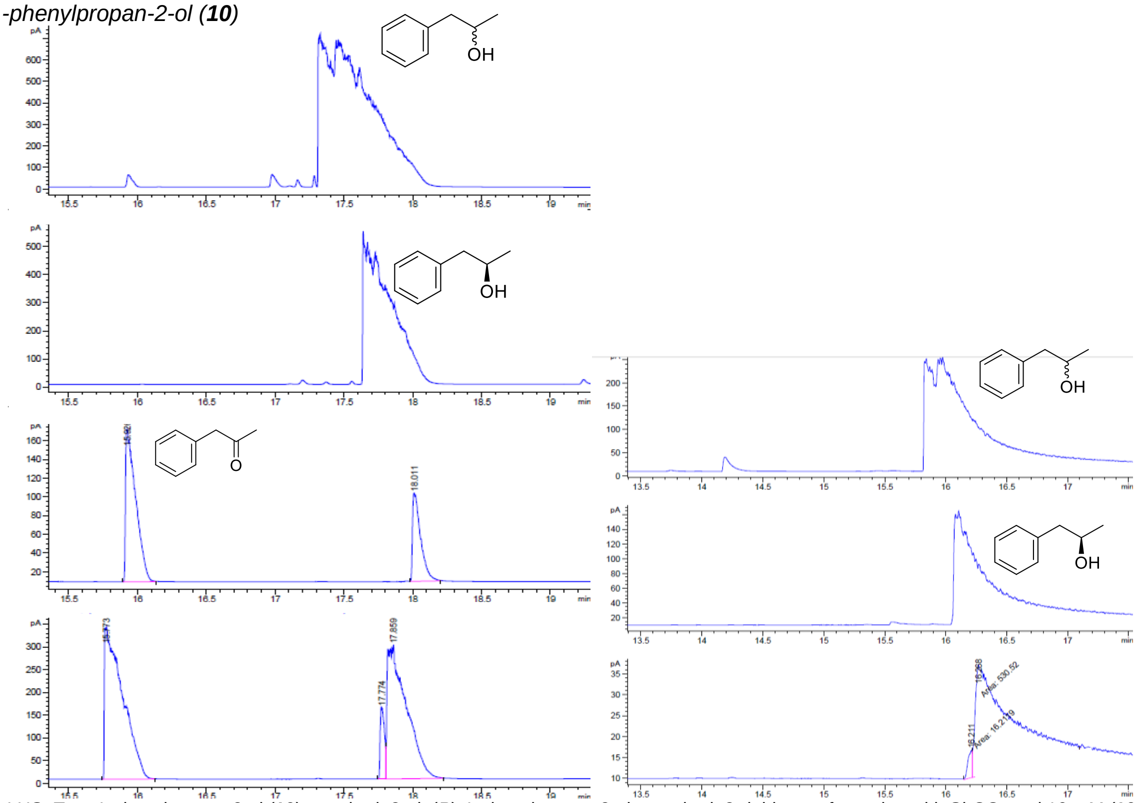

Figure S11: LHS: Top: 1-phenylpropan-2-ol (**10**) standard, 2nd: (*R*)-1-phenylpropan-2-ol standard, 3rd: biotransformation with $ShCO_a$ and 10 mM (**10**), bottom: biotransformation with $ShCO_a$ and 50 mM (**10**). RHS: Top: 1-phenylpropan-2-ol (**10**) standard, 2nd: (*R*)-1-phenylpropan-2-ol standard, 3rd: deracemisation with $ShCO_a$ and 50 mM (**10**) and ammonia borane.

*2.2.3.11 4-phenylbutan-2-ol (**11**)*

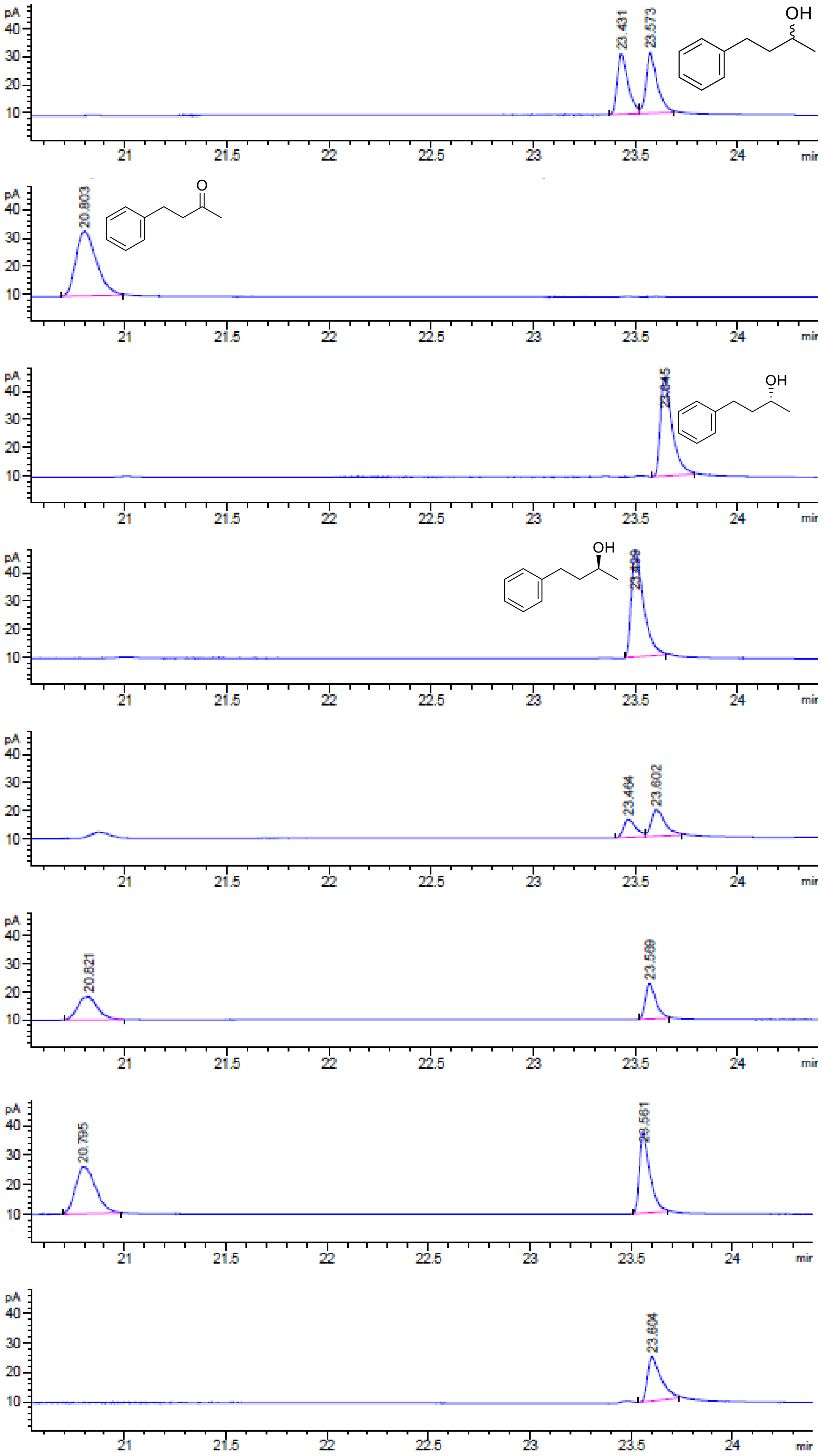

Figure S12: Top: 4-phenyl-2-butanol (**11**) standard; 2nd: 4-phenyl-2-butanone standard; 3rd: (*R*)- 4-phenyl-2-butanol standard; 4th: (*S*)- 4-phenyl-2-butanol standard; 5th: biotransformation with WT and 10 mM (**11**); 6th: biotransformation with ShCO$_b$ and 10 mM (**11**); 7th: biotransformation with ShCO$_b$ and 50 mM (**11**); bottom: deracemisation with ShCO$_b$ and 50 mM (**11**) and ammonia borane. Rt enantiomers = 23.4 min (*S*), 23.6 min (*R*). Rt deracemisation product = 23.6min = (*R*) enantiomer

*2.3.3.12 α-ionol (**12**)*

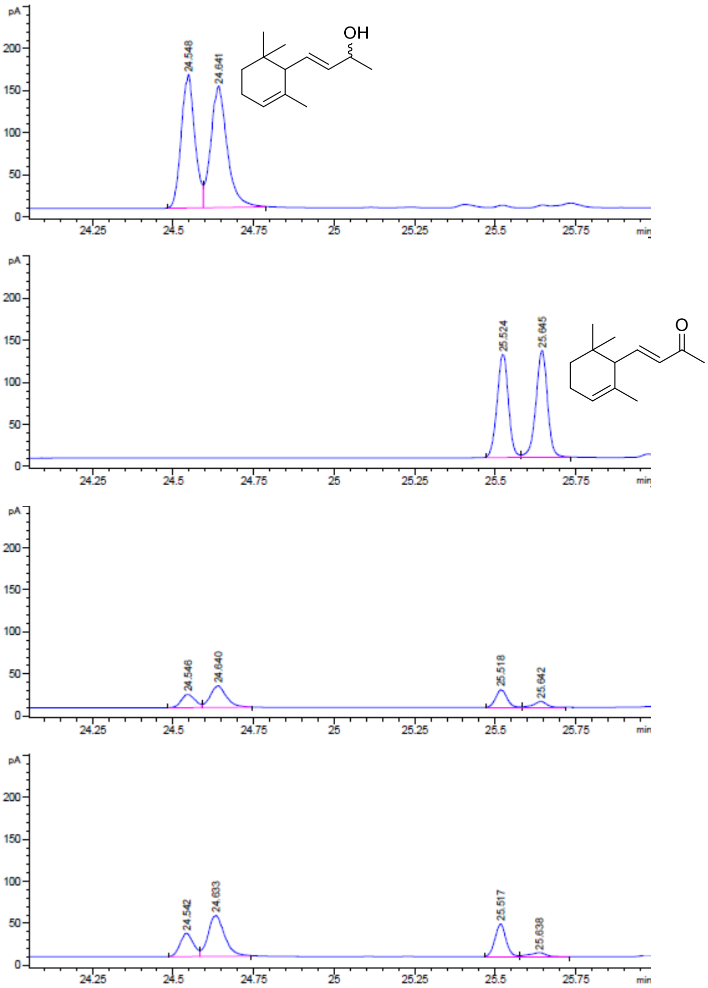

Figure S13: Top: α-ionol (**12**) standard; 2nd: α-ionone standard; 3rd: biotransformation with $ShCO_b$ and 10 mM (**12**); bottom: biotransformation with $ShCO_b$ and 50 mM (**12**).

*2.2.3.13 3-methylbutan-2-ol (**15**)*

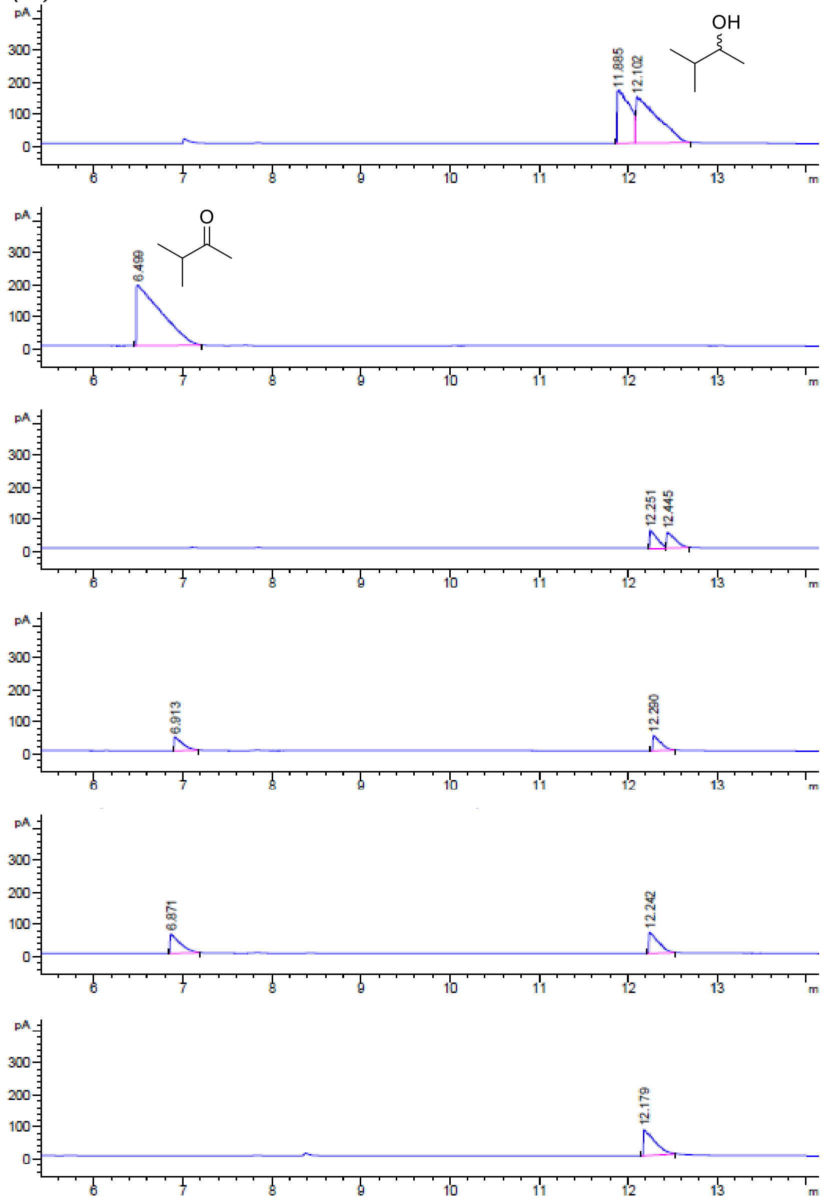

Figure S14: Top: 3-methylbutan-2-ol (**15**) standard; 2nd: 3-methylbutan-2-one standard; 3rd: biotransformation with WTand 10 mM (**15**); 4th: biotransformation with $ShCO_b$ and 10 mM (**15**); 5th: biotransformation with $ShCO_b$ and 50 mM (**15**); bottom: deracemisation with $ShCO_b$ and 50 mM (**15**) and ammonia borane. Rt enantiomers = 12.25 min, 12.45 min, Rt deracemisation product = 12.2 min

*2.2.3.14 2-pentanol (**16**)*

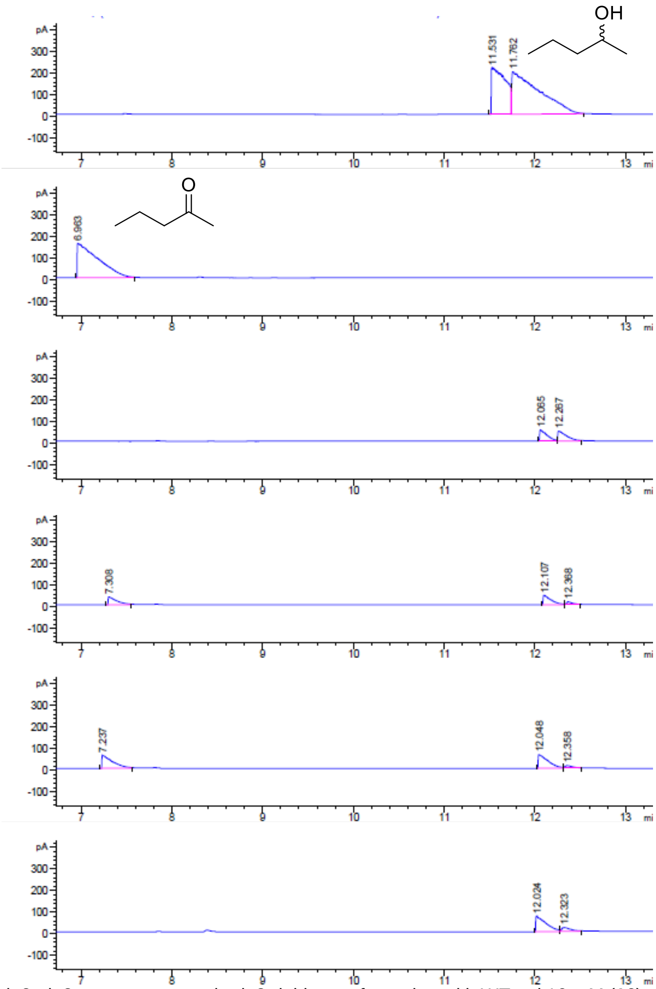

Figure S15: Top: 2-pentanol (**16**) standard; 2nd: 2-pentanone standard; 3rd: biotransformation with WTand 10 mM (**16**); 4th: biotransformation with ShCO$_b$ and 10 mM (**16**); 5th: biotransformation with ShCO$_b$ and 50 mM (**16**); bottom: deracemisation with ShCO$_b$ and 50 mM (**16**) and ammonia borane. Rt enantiomers = 12.1 min, 12.3 min, Rt deracemisation product = 12.1 min (dominant isomer)

*2.2.3.15 1-hexanol (**17**)*

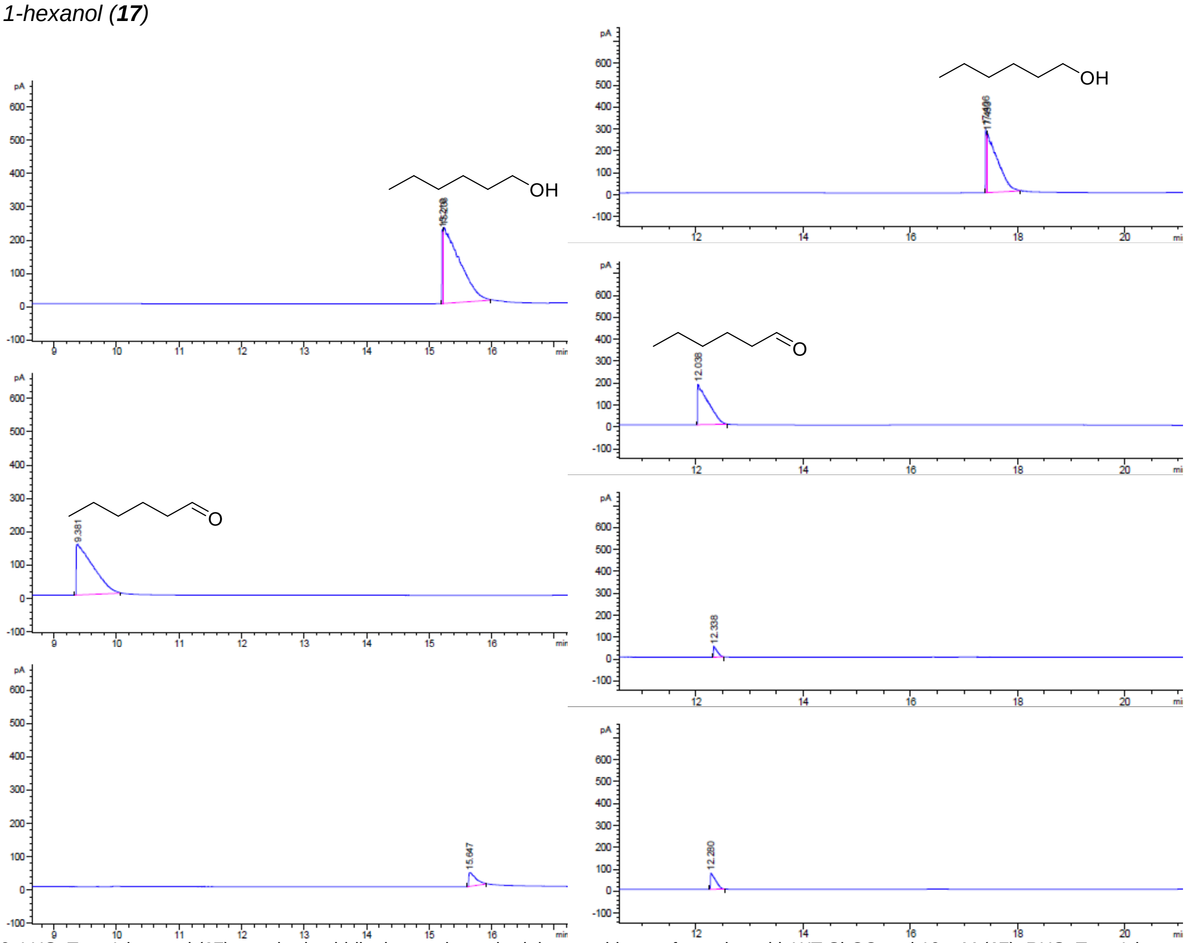

Figure S16: LHS: Top: 1-hexanol (**17**) standard; middle: hexanal standard; bottom: biotransformation with WT ShCO and 10 mM (**17**). RHS: Top: 1-hexanol (**17**) standard; 2nd: hexanal standard; 3rd: biotransformation with ShCO$_b$ and 10 mM (**17**); 4th: biotransformation with ShCO$_b$ and 10 mM (**17**); bottom: biotransformation with ShCO$_b$ and 50 mM (**17**).

2.2.4 100 mg scaled reactions

To 20 mg of purified ShCO variant enzyme in 20 mL air-saturated 100 mM potassium phosphate buffer pH 8.0, in a 200 mL Duran bottle, was added 2 mg catalase (Sigma) and 100 mg of substrate, followed by 20 mL of TBME. The reaction was shaken at 150 rpm, 30 °C overnight. When GC-FID analysis showed complete conversion the product was extracted with 3-fold excess of TBME and dried over magnesium sulphate. TBME was removed under reduced pressure and no further purification was carried out.

2.2.5 NMR and optical rotation (α_D)

^{1}H NMR spectra were recorded on a Bruker Advance 400 (^{1}H at 400.1 MHz) without additional internal standard. Chemical shifts are reported in δ values (ppm) and are calibrated against residual solvent signal.

Optical rotation was recorded on a ADP440 polarimeter (Bellingham + Stanley) against the sodium D-line at 30 °C.

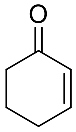

Cyclohex-2-en-1-one

^{1}H NMR (400 MHz, $CDCl_3$) δ = 6.98 (d, *J*=18.3, 1H), 6.42 (td, *J*=31.6, 17.5, 1H), 1.20 (m, 3H), 0.78 (dq, *J*=10.3, 7.5, 7.0, 3H).

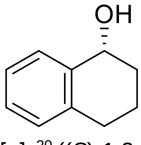

(*R*)-*1,2,3,4-tetrahydronaphthalen-1-ol*
$[\alpha]_D^{20}$ ((*S*)-1,2,3,4-tetrahydronaphthalen-1-ol (Sigma-Aldrich) = +33 ± 1°, c = 2.5% in chloroform[1]
Observed $[\alpha]_D^{30}$ = -35.2°, c = 1.25% in chloroform, thus product is (*R*) enantiomer

^{1}H NMR (400 MHz, $CDCl_3$) δ = 7.36 (m, 1H), 7.13 (m, 2H), 7.03 (m, 1H), 4.72 (dd, *J*=5.7, 3.8, 1H), 2.71 (m, 2H), 1.89 (m, 3H), 1.71 (m, 1H), 1.61 (s, 1H).

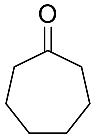

Cycloheptanone
^{1}H NMR (400 MHz, $CDCl_3$) δ = 2.43 (s, 4H), 1.64 (m, 8H).

3. DNA and protein methods

3.1 Protein sequences

Rhodococcus erythropolis
MSGVKRRSFLAGTAAVGVAMMAPRTAKAATGIPLTREVHRVVVIGSGFGGGVTALRLAEAGVPVTLLERGIRWQTGPNAETFPHPAS
PDKRILWHQSNPTLFGRPAIFEPYAGLLETVTGENMTAICAAGVGGGSLVYQGMTLQPEEAVFNTHFPEELDYARMDRVHYPRVARM
LQVETAPDELISSPNYEAARVFARNATRSGLPVSKIPMPIDWNYALAELRGEMKPSYTNGDGALGVNNGGKHSVDVTYIAAAEATGLV
NVETLHQVTDVERAADGRWRVYVDRTDISGKILENKILTTDALVMAAGSMNTTKLLVRAAATGRITDLPDELGAGWGTNADRIYIWSDL
AENFGATQGGPVVYGSRNWDDPHNAFTVIQASFPPVAFDAHSTVLVGFGVSSDRGRFVYDSARGEAVLRWPRDGDSAIQQGHIGPA
AQRIAAGGLLTDTNALLPSTWHPLGGACMNSVCDLDGRVLGQRGLYVLDGALLPGNSAACNPSMTIAAVAERALENIVKDDVGTLF
Brevibacterium sterolicum
MSTGPVAPLPTPPNFPNDIALFQQAYQNWSKEIMLDATWVCSPKTPQDVVRLANWAHEHDYKIRPRGAMHGWTPLTVEKGANVEKVI
LADTMTHLNGITVNTGGPVATVTAGAGASEAIVTELQKHDLGWANLPAPGVLSIGGALAVNAHGAALPAVGQTTLPGHTYGSLSNLVT
ELTAVVWNGTTYALETYQRNDPRITPLLTNLGRCFLTSVTMQAGPNFRQRCQSYTDIPWRELFAPKGADGRTFEKFVAESGGAEAIWY
PFTEKPWMKVWTVSPTKPDSSNEVGSLGSAGSLVGKPPQAREVSGPYNYIFSDNLPEPITDMIGAINAGNPGIAPLFGPAMYEITKLGL
AATNANDIWGWSKDVQFYIKATTLRLTEGGGAVVTSRANIATVINDFTEWFHERIEFYRAKGEFPLNGPVEIRCCGLDQAADVKVPSVG
PPTISATRPRPDHPDWDVAIWLNVLGVPGTPGMFEFYREMEQWMRSHYNNDDATFRPEWSKGWAFGPDPYTDNDIVTNKMRATYIE
GVPTTENWDTARARYNQIDPHRVFTNGFMDKLLP
Streptomyces hygrospinosus
MFENQQNQHLSRRRLLGLAALSGAAVTGLTTISAAPRAAAADKRSPRADSGSFVPAVVIGTGYGAAVSALRLGEAGVETLMLEMGQL
WNKPAEDGNVFCGMLTPDRRSSWFKSRTEAPLGSFLWLDVINRDIEPYAGVLDRVHFDQMSVYVGRGVGGGSLVNGGMAVVPKRA
YFEEVLPQVDAAQMYERYFPRANAALKVNHIDPAWFEKTEWYNFARVSREQAGKAGLSTTFVPNVYDFDHMQREAAGTAPKSALAG
EVIYGNNHGKQSLDKTYLAAALGTGKVTIETLHRVTAIRQQADGSYVLSVDQSDANGTVIAHKEIACRHLFLGAGSLGSTELLVRARDTG
ALPHLNAEVGEGWGPNGNIMTGRANHVWNPTGAHQSSIPALGIDDWDNPDAPVFAEIAPMPAGLETWVSLYLAITKNPQRGSFVYDK
ATDRAMLRWTREQNAPAVAAAKSLFDRINKANTTMYRYDLFGPQLKNFADDFCYHPLGGCVLGKATDDYGRVAGYHNLYVTDGALIP
GSIGVNPFVTITALAERNIERVIAEDVRTAA

3.2 Cloning

The gene for cholesterol oxidases from *Streptomyces hygrospinosus, Brevibacterium sterolicum and Rhodococcus erythropolis* were codon optimised for expression in *E. coli* and synthesised by GeneArt® (Life Technologies). The gene was sub-cloned into the vector pET28b (also digested with NdeI/XhoI and then CIP (New England Biolabs (NEB)) to remove phosphates) following the protocol in the Quick ligation kit (NEB) and transformed into *E. coli* NEB10β (NEB). Clones were sequenced at MWG Eurofins using T7 and T7term primers to verify the insert and then transformed into *E. coli* BL21 (DE3) (NEB) for expression.

3.3 Expression and purification

Overnight cultures were prepared by inoculating 6 mL of LB (complemted with 50 µg / mL kanamycin) with a single colony and grown for 16 hours at 37 °C at 200 rpm. The overnight culture was then added to a 2 L flask containing 600 mL autoinduction media (LB based with trace elements (Formedium)) and 50 µg / mL kanamycin and grown at 14 °C with shaking at 200 rpm for 96 hours. Cells were harvested by centrifutgation at 4000 rpm for 20 min and stored as pellets at -20 °C. Purification was carried out as in reference 2.[2]

3.4 Libraries

3.4.1 Synthesis of libraries
Saturated libraries were made by designing primers at the appropriate regions in the *Streptomyces hygrospinosus* cholesterol oxidase gene and following the protocol from the QuikChange II Site-Directed Mutagenesis Kit (Agilent Technolgies Pfu polymerase was replaced with Phusion polymerase (NEB) and dNTPs replaced with dNTPs from NEB. Primers were synthesised by MWG Eurofins. Primer sequences are shown below in Table S2. N means any base and S = C/G.

Table S2: Primer sequences

Name	Sequence
L418L420_F	ACCTGGGTTAGCNNSTATNNSGCAATTACCAAAAATCC
L418L420_R	GGATTTTTGGTAATTGCSNNATASNNGCTAACCCAGGT
P387_F	CAGAGCAGCATTNNSGCACTGGGTATTG
P387_R	CAATACCCAGTGCSNNAATGCTGCTCTG
F122 _F	CGCTGGGTAGCNNSCTGTGGCTGGATG
F122_R	CATCCAGCCACAGSNNGCTACCCAGCG
F476_F	CGCTATGACCTGNNSGGTCCGCAGCTG
F476_R	CAGCTGCGGACCSNNCAGGTCATAGCG
M165_F	GGTGAATGGTGGTNNSGCCGTTGTTCCG
M165_R	CGGAACAACGGCSNNACCACCATTCACC
E404/P409 _F	CCGGTTTTTGCCNNSATTGCACCGATGNNSGCAGGTCTGG
E404/P409_R	CCAGACCTGCSNNCATCGGTGCAATSNNGGCAAAAACCGG
Y489/H490_F	GCAGATGATTTTTGTNNSNNSCCGCTGGGTGGTTGTG
Y489/H490_R	CACAACCACCCAGCGGSNNSNNACAAAAATCATCTGC
N528_F	GGTAGCATTGGTGTTNNSCCGTTTGTTACC
N528_R	GGTAACAAACGGSNNAACACCAATGCTACC

3.4.2 Solid phase screen

Libraries were transformed into *E. coli* BL21 (DE3) (NEB) according to the manufactureres instructions, except that the whole transformation reaction was plated on top of a membrane (HyBond) on LB agar containing 50 µg mL-1 kanamycin and grown overnight at 30 °C. The membrane was then transferred to a second LB agar plate (containing 50 µg mL^{-1} kanamycin and 1 mM IPTG) for induction of prottein expression. After six hours at 20 °C the membranes were transferred to petri dishes and stored at -20 °C until use. To perform the screen, filter paper containing 0.1 mg mL-1 Horse-radish peroxidase (HRP) (Sigma) in pH 8.0, 100 mM phosphate buffer was prepared. The membranes were freeze-thawed three times (using liquid N_2) in order to partially lyse the colonies and were then placed on the filter paper. After one hour at room temperature (to ensure removal of any cellular H2O2) the membrane was transferred to another filter paper which had been soaked in assay solution. (The assay solution consited of of 0.1 mg mL-1 HRP, 3,3'-Diaminobenzidine (DAB) (1 tablet per 15 mL, SigmaFast, Sigma), and 100 mM substrate in 100 mM potassium phosphate buffer, pH 8.0.). A positive hit was indicated by colonies that changed to a dark red/brown colour. These colonies were culture and the plasmid DNA extracted and sequenced using T7 or T7 terminator primers (MWG Eurofins).

3.5 Kinetics

Cholesterol oxidase produces hydrogen peroxide as it turns over. Hydrogen peroxide production can be detected by horse-radish peroxidase (Type VI, Sigma) and the dye ABTS (Sigma) (ε = 36000 L mol^{-1} cm^{-1}). The assay was carried out in a 96 well assay plate to which was added 50 µL substrate (dissolved in DMSO to 1 M then diluted in buffer), 50 µL ABTS (0.7 mg mL^{-1}) and 50 µL HRP (0.4 mg mL^{-1}). The assay was started by adding 50 µL purified enzyme (typically 0.1 – 0.3 mg mL^{-1}). Initial rates were calculated by following the absorbance at 420 nm over time on a TECAN Infinite M200 spectrophotometer at 30 °C. Typically, eight different substrates concentrations were examined (typically 250 mM down to 0.5 mM). A plot of rate vs substrate concentration (Figure S17) allowed the extraction of V_{max} and K_M values using a non-linear curve fit analysis using the Hill model (OriginPro 9.1). k_{cat} and K_M error values represent a 95 % confidence interval based on the standard error of the regression.

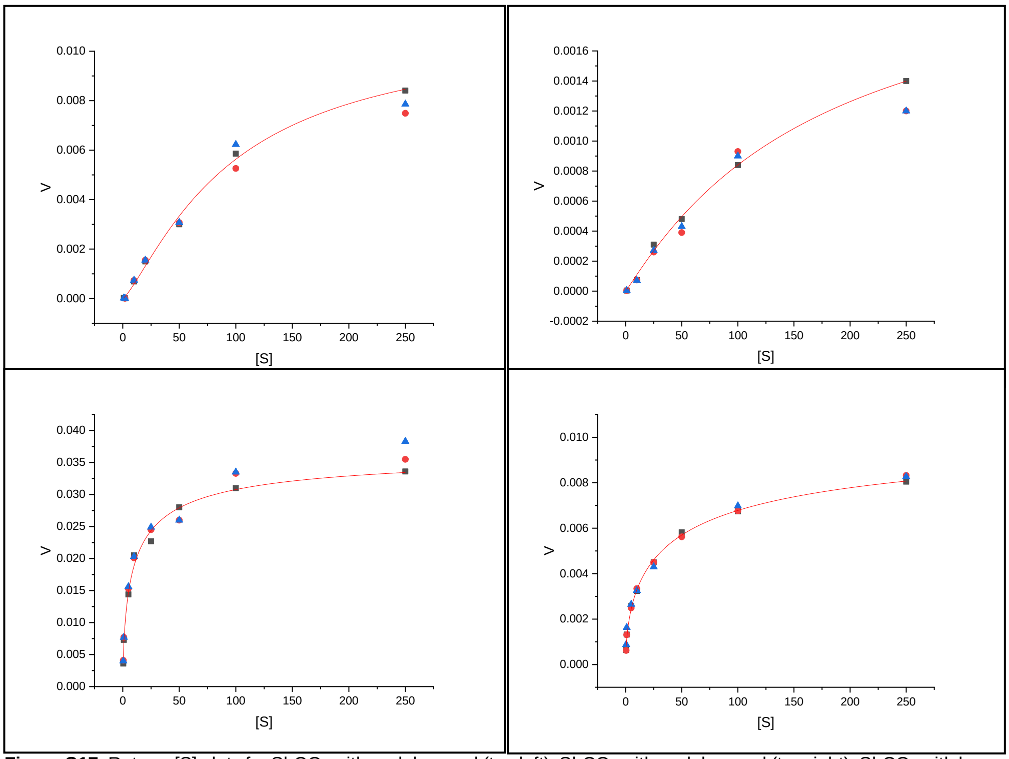

Figure S17: Rate vs [S] plots for ShCO$_b$ with cyclohexanol (top left), ShCO$_a$ with cyclohexanol (top right), ShCO$_b$ with hexanol (bottom left), ShCO$_a$ with cyclohexanol (bottom right)

3.6 Temperature and solvent stability

3.6.1 Biotransformations using solvent overlays

500 µl biotransformation reactions were set up (final concentration enzyme: 1 mg ml^{-1}, final concentration 2-cyclohexen-1-ol: 90 mM) and 500 µl of solvent was overlayed. Biotransformations were incubated at 30°C, 180 rpm for 24 hrs. Extractions were carried out by addition of a further 500 µl of the same solvent and extraction and analysis continued as described in 2.1 and 2.2.1 using GC-FID method B.

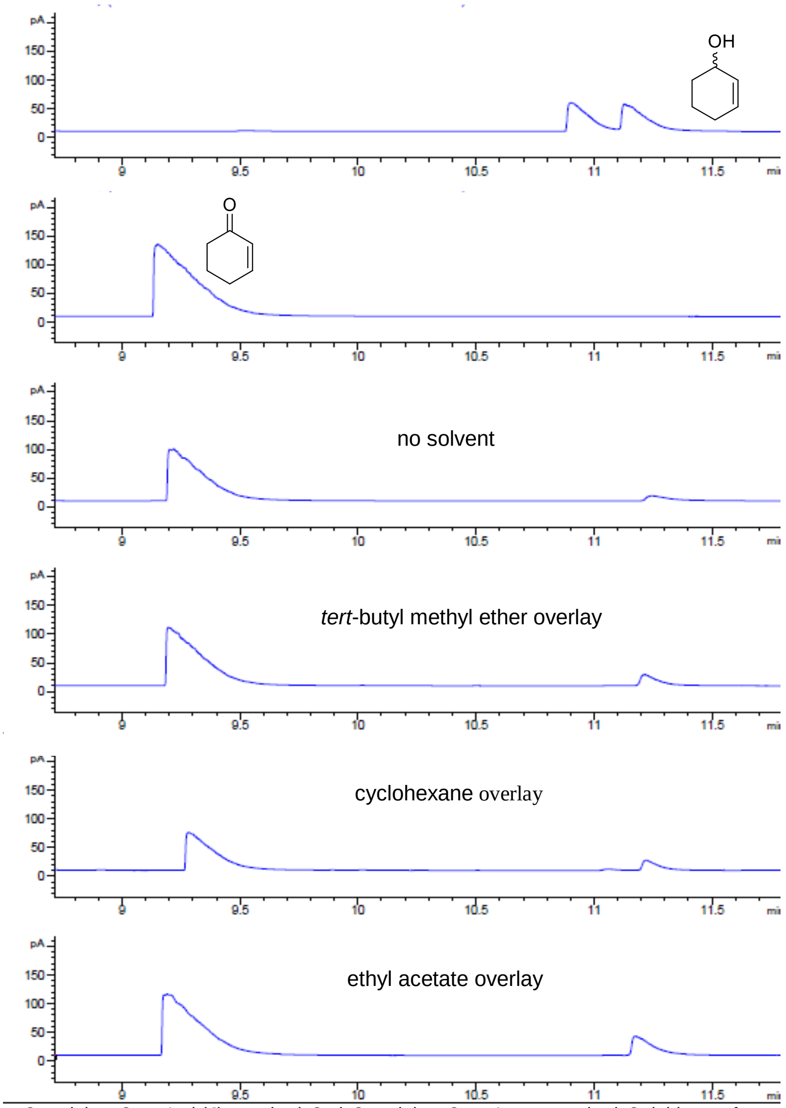

Figure S18: Solvent tolerance: Top: 2-cyclohex-2-en-1-ol (**4**) standard, 2nd: 2-cyclohex-2-en-1-one standard, 3rd: biotransformation with no solvent (**4**), 4th: biotransformation with *tert*-butyl methyl ether overlay, 5th: biotransformation with cyclohexane overlay, bottom: biotransformation with ethyl acetate overlay.

3.6.2 T_{50} calculations

Purified enzyme was incubated at the given temperature for 15 minutes. An initial rates experiment was then conducted with 100 mM hexanol (see 3.5 for assay components) and the highest initial rate for each variant was set to 100%. Other rates were then compared to this to give a relative activity. A sigmoidal dose-response (variable slope) non-linear fit was applied in GraphPad Prism.

3.7 Modelling

A homology model of Streptomyces hygrospinosus cholesterol oxidase was prepared using YASARA (www.yasara.org).[3]

Results and Discussion

Hits from screening libraries

The hits from each library are described in Table S3 with the most active variant confirmed in the right-hand column.

Table S3: Hits from library screening

Screening substrate	Library screened	Hits	Most active variant
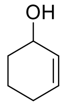	E404/P409	E404C/P409S, E404C/P409L	E404C/P409S = $ShCO_a$
	P387	P387W	
	L418/L420	L418A/L420F	
	F122	No hits	
	Y489/H490	No hits	
	N528	No hits	
	M165	No hits	
	F476	No hits	
	E404C/P409S/L418A/L420F/P387[a]	No hits	
	E404C/P409S/P387 [a]	No hits	
	L418A/L420F/P387 [a]	No hits	
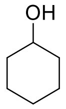	E404/P409	E404N/P409L, E404N/P409C, E404A/P409I	E404A/P409I = $ShCO_b$
	L418/L420	No hits	
	P387	No hits	
	F122	F122C	
	M165	No hits	
	N528	No hits	
	E404N/P409L/F122 [a]	No hits	
	F476	No hits	
	Y489/H490	No hits	

[a]These libraries were constructed because we found hits in the initial libraries and proposed that we could gain better activity by taking an iterative approach where one library is made in the background of a variant that is already active.

Additionally we combined E404C/P409S and P387W to make a three point variant but activity with 2-cyclohexen-1-ol went from full conversion (with E404C/P409S) to 25% conversion. A four point variant E404C/P409S/L418A/L420F was constructed and conversion went from full conversion (E404C/P409S) to 65% with 2-cyclohexen-1-ol although it was apparent that the enzyme was selective. F122C was combined with E404N/P409L but activity remained the same. Activities were comparable between E404N/P409L, and E404A/P409I on cyclohexanol but E404A/P409I showed better convesrions with the other substrates.

Kinetics of variants with hexanol and cylcohexanol

The kinetics of the variants were established with hexanol and cyclohexanol (Table S4)

Table S4: Kinetics

Substrate	Enzyme	k_{cat} s^{-1}	K_M / mM
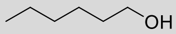	wt	n.m.	n.m.
	$ShCO_a$	0.69±0.06	46.8±17.1
	$ShCO_b$	1.86±0.12	9.7±2.6
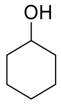	wt	n.m.	n.m.
	$ShCO_a$	0.14±0.03	175.7±71.5
	$ShCO_b$	0.54±0.06	95.8±19.6

n.m – activity too low to be measurable

For comparison, a homologous *Streptomyces* cholesterol oxidase has a k_{cat} of 44±2 s^{-1} and a K_M of 3±0.4 µM with the natural substrate cholesterol.[4]

Modelling of inactive substrate
Modelling of substrate **13** (Figure S19) in the active site shows a potential clash with the FAD cofactor, suggesting why **13** is not a good substrate.

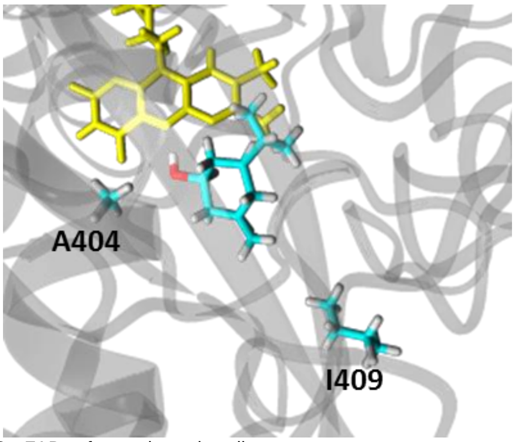

Figure S19: Model of **13** in the active site of $ShCO_b$, FAD cofactor shown in yellow.

Temperature stability
The $LogEC_{50}$ values returned (in this case the temperature at which 50% activity remained) were defined as the T_{50} for the enzyme of 50.9 ± 0.5 °C. (Figure S20).

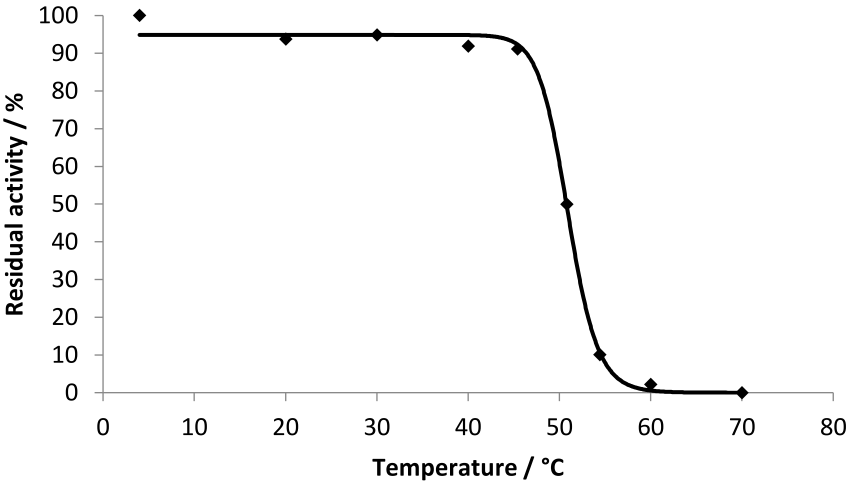

Figure S20: Non-linear fit to data from temperature stability experiments using GraphPad Prism

References

[1] sds on https://www.sigmaaldrich.com/catalog/product/sial/87649, accessed 15/12/2020
[2] Heath, R. S., Birmingham, W. R., Thompson, M. P., Taglieber, A., Daviet, L., Turner, N. J., *ChemBioChem* **2019**, 20, 276
[3] Krieger E, Vriend G., *Bioinformatics.* **2014**, 30, (20), 2981-2982
[4] Yue, Q. K., Kasss, I. J., Sampson, N. S., Vrielink, A. *Biochemistry* **1999**, 38, 14, 4277–4286

Author Contributions

RSH designed and performed the experiments and analysis and wrote the manuscript, JJS performed analytical experiments and NJT wrote the manuscript and acquired the funding.
